# Genomic aberrations in cell cycle genes predict progression of *KIT*-mutant gastrointestinal stromal tumors (GISTs)

**DOI:** 10.1186/s13569-019-0112-7

**Published:** 2019-03-05

**Authors:** Michael C. Heinrich, Janice Patterson, Carol Beadling, Yuexiang Wang, Maria Debiec-Rychter, Barbara Dewaele, Christopher L. Corless, Anette Duensing, Chandrajit P. Raut, Brian Rubin, Tamas Ordog, Matt van de Rijn, Jerry Call, Thomas Mühlenberg, Jonathan A. Fletcher, Sebastian Bauer

**Affiliations:** 1 Hematology/Medical Oncology, Portland VA Health Care System and OHSU Knight Cancer Institute, 3710 SW U.S. Veterans Hospital Road, R&D 19, Portland, OR 97239 USA; 2 Department of Pathology, Brigham and Women’s Hospital, 75 Francis Street, Boston, MA 02115 USA; 3 Department of Human Genetics, Katholieke Universiteit Leuven and University Hospitals Leuven, Herestraat 49, 3000 Leuven, Belgium; 4 Cancer Therapeutics Program, UPMC Hillman Cancer Center, 5117 Centre Avenue, Pittsburgh, PA 15213 USA; 5 Department of Surgery, Brigham and Women’s Hospital, Dana-Farber Cancer Institute and Harvard Medical School, Boston, MA USA; 6 Department of Molecular Genetics, Cleveland Clinic and Lerner Research Institute, L25, 9500 Euclid Avenue, Cleveland, OH 44195 USA; 7 Department of Physiology and Biomedical Engineering, Division of Gastroenterology and Hepatology and Center for Individualized Medicine, Mayo Clinic, 200 1st Street SW, Rochester, MN USA; 8 Department of Pathology, Stanford University Medical Center, 300 Pasteur Dr., Stanford, CA 94305 USA; 9 The Life Raft Group, 155 Route 46 West, Suite 202, Wayne, NJ 07470 USA; 10 Department of Medical Oncology, West German Cancer Center, University Hospital Essen, University of Duisburg-Essen, Hufelandstrasse 55, 45147 Essen, Germany; 11 Germany and German Cancer Consortium (DKTK), Partner Site University Hospital Essen, Essen, Germany

**Keywords:** GI stromal tumor, Sarcoma, Cell cycle, KIT, TP53, RB1, CDKN2A, Spliceosome

## Abstract

**Background:**

Activating mutations of the receptor tyrosine kinase *KIT* are early events in the development of most gastrointestinal stromal tumors (GISTs). Although GISTs generally remain dependent on oncogenic *KIT* during tumor progression, *KIT* mutations alone are insufficient to induce malignant behavior. This is evidenced by *KIT*-mutant micro-GISTs, which are present in up to one-third of normal individuals, but virtually never progress to malignancy.

**Methods:**

We performed whole exome sequencing on 29 tumors obtained from 21 patients with high grade or metastatic *KIT*-mutant GIST (discovery set). We further validated the frequency and potential prognostic significance of aberrations in *CDKN2A/B, RB1,* and *TP53* in an independent series of 71 patients with primary GIST (validation set).

**Results:**

Using whole exome sequencing we found significant enrichment of genomic aberrations in cell cycle-associated genes (Fisher’s Exact p = 0.001), most commonly affecting *CDKN2A/B, RB1, and TP53* in our discovery set. We found a low mutational tumor burden in these 29 advanced GIST samples, a finding with significant implications for the development of immunotherapy for GIST. In addition, we found mutation of spliceosome genes in a minority of cases, implicating dysregulation of splicing as a potential cancer promoting mechanism in GIST. We next assessed the prognostic significance of *CDKN2A, RB1* or *TP53* mutation/copy loss in an independent cohort of 71 patients with primary GIST. Genetic events (mutation, deletion, and/or LOH) involving at least one of the three genes examined were found in 17% of the very low-risk, 36% of the low-risk, 42% of the intermediate risk, 67% of the high-risk/low mitotic-count, and in 86% of the high-risk/high mitotic-count group. The presence of cell cycle-related events was associated with a significantly shorter relapse-free survival (median 67 months versus not reached; *p* < 0.0001) and overall survival (Log Rank, *p *= 0.042).

**Conclusion:**

Our results demonstrate that genomic events targeting cell cycle-related genes are associated with GIST progression to malignant disease. Based on this data, we propose a model for molecular pathogenesis of malignant GIST.

**Electronic supplementary material:**

The online version of this article (10.1186/s13569-019-0112-7) contains supplementary material, which is available to authorized users.

## Introduction

Gastrointestinal stromal tumors (GISTs) are the most common mesenchymal tumors of the gastrointestinal tract. The vast majority of these tumors have activating mutations of the *KIT* or *PDGFRA* receptor tyrosine kinases that are considered to be initiating oncogenic events [1, 2]. GISTs lacking *KIT* or *PDGFRA* mutations comprise 10–15% of the cases. Alternative oncogenic events in these tumors include activating mutations of *BRAF* or *KRAS* or inactivating mutations of NF1 or genes encoding the succinate dehydrogenase (SDH) complex [3–5]. The oncogenic reliance of GISTs upon mutated *KIT/PDGFRA* is emphasized by their sensitivity to imatinib mesylate (IM), a tyrosine kinase inhibitor (TKI) that targets both KIT and PDGFRA kinase activity, leading to substantial tumor shrinkage with durable responses in most patients [6, 7].

However, IM is not a cure for GIST. Most patients will eventually experience tumor progression despite continuous IM treatment [8, 9]. Resistance to IM often arises due to secondary mutations within *KIT* or *PDGFRA*. Such mutations are commonly found in tumors removed from patients with clinical IM resistance. Additional kinase inhibitors target these secondary mutations and are used as second-line or later treatment for IM-resistant GISTs [10, 11]. Nevertheless, the progression-free survival with these salvage tyrosine kinase inhibitors is limited, and most patients experience progression from these agents within 6–12 months [6, 12].

Numerous studies have reported small incidental GISTs, less than a centimeter in diameter, that are identified during gastrectomy or at autopsy [13–16]. Many of these lesions harbor *KIT* mutations identical to those identified in larger malignant lesions, indicating that additional genetic events are necessary to transform these micro-GISTs into malignant tumors. Detection of genomic events associated with the development of malignant GISTs could therefore identify patients at high risk of recurrence after curative-intent resection of a primary tumor. Here we report that genomic alterations in key cell cycle regulators are recurrent abnormalities in patients with advanced GIST.

## Methods

### Patients and tumor tissues

De-identified samples analyzed by whole exome sequencing (WES) were obtained from participating institutions (Oregon Health and Science University, University of Duisburg-Essen, Brigham and Women`s Hospital and Katholieke Universiteit Leuven and University Hospitals Leuven). This study was approved by the institutional review boards at each of the participating sites. For whole exome sequencing approach, we selected 21 patients with high-risk GISTs, including 20 patients who presented with or eventually developed metastatic disease (discovery set). All tumor samples were obtained from frozen tissues except for one (19A), which was obtained from formalin-fixed paraffin embedded (FFPE) tissue. Paired normal DNA was extracted from frozen normal tissue (32%, 7/22), blood (63%, 14/22), or FFPE tissue (5%, 1/22).

Targeted sequence analysis was performed on a second group of 71 GIST samples representing an independent cohort of clinically characterized patients from prospectively maintained databases of participating institutions (University of Duisburg-Essen, Brigham and Women`s Hospital, and Oregon Health and Science University) and from the Life Raft Group patient database (validation data set) [17]. This cohort study was approved by the institutional review boards at each of the participating sites.

Progression-free survival (PFS) was calculated as the length of time from diagnosis of localized disease to the date of documented recurrence or death from any cause, whichever occurred first. Overall survival (OS) was defined as the length of time from the time of diagnosis of localized disease to death from any cause. PFS and OS estimates and standard errors determined using the Kaplan–Meier method and statistical comparisons were performed using the log-rank test. Two-sided p values less than 0.05 were considered to be statistically significant. The statistical analyses were performed using SPSS 19.0 and R 2.15.2 (http://www.r-project.org/). We conducted a multivariate analysis for PFS using three covariates (mitotic count, size and cell-cycle-related genomic events) using SAS software 9.4 (SAS Institute Inc., Cary, NC, USA).

### Whole exome sequencing

The experimental and computational details of these experiments are included in additional methods.

### Ion torrent targeted-exome genomic sequencing

In an independent validation cohort of 71 patients, targeted sequence analysis was performed with a custom AmpliSeq panel (Life Technologies, Grand Island, NY) that includes 24 genes (AKT1, AKT2, AKT3, ATM, BRAF, CDKN2A, HRAS, KIT, KRAS, MAP2K1, NF1, NRAS, PDGFRA, PIK3CA, PTEN, PTPN11, RB1, SDHA, SDHAF1, SDHAF2, SDHB, SDHC, SDHD, TP53). Sequencing was carried out on an Ion Torrent PGM instrument (Thermo Fisher Scientific, Inc., Waltham, MA), and Torrent Suite Software v3.2 was used for sequence alignment and variant calling.

### Interphase FISH on tumor sections

To detect deletions affecting the CDKN2A/B/9p21.3, RB1/13q14 and TP53/17p13 genes, cut sections (4 µm thick) from paraffin-embedded tumor tissues from 42 patients were subjected to separate interphase FISH assays. FISH assays were performed using the commercially available dual-color probes LSI CDKN2A/B (9p21)-Spectrum Orange (SO)/CEP9-Spectrum Green (SG), LSI RB1-SO/CEPX-SG and LSI TP53-SO/CEP17-SG cocktails (all from Abbott Laboratories, Des Plaines, IL). Probe hybridization and detection were carried out according to standard methods. Slides were analyzed blinded from the clinical data, using a Zeiss microscope (Axioplan 2, Jena, Germany) equipped with the appropriate filters. The numbers of differentially labeled hybridization signals representing the investigated gene and reference centromere chromosomal regions were individually recorded for 100 non-overlapping interphase nuclei in at least two different areas of the section. Zero to 1 green centromeric (green) signals per nucleus in > 60% of cells were defined as a whole chromosome loss. In addition, the ratio of red to green signals was calculated. Heterozygous or homozygous losses were delineated by ratios < 0.6 and < 0.3, respectively.

### Multiplex ligation-dependent probe amplification (MLPA)

Gene dosage ratios for 12 *CDKN2A/CDKN2B* loci and 11 other chromosome 9p genes were determined using the 9p21 MLPA kit (ME024-B1 MRC-Holland, Amsterdam, Netherlands). The probe-mix contains 21 different probes for the *CDKN2A/B* genes. In addition, it contains 2 probes each for *MTAP, CDKN2B*-*AS1, PAX5 and MIR31*, and 4 probes between the *MIR31* gene and the 9p telomere. Two digestion control probes and 12 reference probes were included for data analysis. In each set of experiments, one negative control sample (no DNA) and 6 normal control samples (DNA isolated from paraffin sections of normal human intestines from different individuals) were included. Briefly, 50 ng of extracted tumor DNA was denatured and target gene probes were hybridized to the target DNA prior to probe ligation in the presence of Ligase-65 (MRC-Holland). The ligation products were subjected to polymerase chain reaction (PCR) amplification performed on a GeneAmp PCR System 9700 Thermal Cycler (Applied Biosystems, Warrington, UK) with a hot-start PCR program. MLPA fragments were visualized on an ABI 3130XL Automated DNA Sequencer (Applied Biosystems). Peak detection analysis has been automated using ABI PRISM Genescan^®^ Analysis software version 3.1 (Applied Biosystems) and GeneMarker software (Softgenetics, State College, PA, USA).

### CDKN2A copy number variation assessment using SNP arrays

CDKN2A homozygous deletions were identified by Affymetrix 250 K Nsp1 SNP arrays (Thermo Fisher Scientific). High-molecular-weight genomic DNA was isolated using the QIAamp DNA Mini kit (Qiagen). DNA was digested with Nsp1, and linkers were ligated to the restriction fragments to permit PCR amplification. PCR products were purified and fragmented by treatment with DNase I, and fragments were then labeled and hybridized to microarray chips. The positions and intensities of fluorescence emissions were analyzed using Chip software. Array intensity values were normalized to the value for the array with median intensity.

## Results

### Clinical characteristics of GIST patients analyzed by whole exome sequencing (discovery set)

We analyzed 29 high-risk GIST lesions paired with normal tissue obtained from 21 patients (Discovery Set, Table 1). Twenty of these patients presented with, or developed, metastatic disease, and thirteen of these patients had one to ten metastatic lesions that were surgically removed after clinical resistance to one or more tyrosine kinase inhibitors (median of one lesion). Seventeen of the 21 patients had tumor samples collected after one or more lines of TKI therapy. Secondary KIT mutations were identified in 18 of the 25 tumors harvested after TKI therapy (72%). In the other seven tumors, only a primary *KIT* gain-of-function mutation was identified. As expected, only a primary KIT mutation was found in samples from the four patients whose tumors were collected prior to TKI therapy.

**Table 1:** Clinicopathological features of the discovery set (21 patients, 29 samples)

	ID	Genotype	Gender	Age at Diagnosis	Site of Primary Tumor	Sample type	Metastatic disease	Therapies prior to sample harvest
1	1A	KIT exon 9 insertion AY502–503; exon 13 N655S; exon 17 N822 K	Male	45	Small intestine	Metastasis	Y	IM, SU
	1B	KIT exon 9 insertion AY502–503; exon 17 N822K	Male	45	Small intestine	Metastasis	Y	IM, SU
	1C	KIT exon 9 insertion AY502–503, exon 13 N655S; exon 17 N822K	Male	45	Small intestine	Metastasis	Y	IM, SU
2	2A	KIT exon 9 insertion AY502–503; exon 17 D816H	Male	56	Small intestine	Metastasis	Y	IM, SU
	2B	KIT exon 9 insertion AY502–503; exon 17 D820E	Male	56	Small intestine	Metastasis	Y	IM, SU
3	3A	KIT exon 11 deletion W557-V559	Male	60	Small intestine	Metastasis	Y	IM
4	4A	KIT exon 11 deletion VQWKV 555–559; exon 13 V654A	Male	50	Stomach	Metastasis	Y	IM
5	5A	KIT exon 9 insertion AY502–503; exon 17 Y823D	Female	33	Small intestine	Metastasis	Y	IM, SU, NI, SO, IM + LBH, DA
	5B	KIT exon 9 insertion AY502–503; exon 17 D820E; exon 18 S840 N	Female	33	Small intestine	Metastasis	Y	IM, SU, NI, SO, IM + LBH, SO + RAP, DA, DOX + GEM, PA
6	6A	KIT exon 11 deletion D579	Male	65	Small intestine	Metastasis	Y	IM
7	7A	KIT exon 11 deletion W557-K558	Female	39	Small intestine	Metastasis	Y	IM
8	8A	KIT exon 11 V559D	Male	65	Rectum	Primary tumor	Y	IM
9	9A	KIT exon 11 deletion K550-K558; KIT exon 17 D820G	Male	65	Stomach	Primary tumor	Y	IM
10	10A	KIT exon 11 deletion K550–558 (starts intron 10); exon 17 D820Y	Female	43	Small intestine	Metastasis	Y	IM, SU, RE
	10B	KIT exon 11 deletion K550–558 (starts intron 10); exon 17 D820Y	Female	43	Small intestine	Metastasis	Y	IM, SU, RE
11	11A	KIT exon 11 deletion E554-D572; exon 13 V654A	Male	45	Small intestine	Metastasis	Y	IM
12	12A	KIT exon 11 deletion N567-L576	Male	56	Small intestine	Metastasis	Y	IM
13	13A	KIT exon 9 S476I	Male	40	Small intestine	Metastasis	Y	IM, SU
14	14A	KIT exon 11 deletion G554-V559; Exon 17 N822 K	Male	28	Small intestine	Metastasis	Y	IM, SU, NI, SO, DA
	14B	KIT exon 11 deletion G554-V559; Exon 17 N822 K	Male	28	Small intestine	Metastasis	Y	IM, SU, NI, SO, DA
	14C	KIT exon 11 deletion G554-V559; Exon 17 N822 K	Male	28	Small intestine	Metastasis	Y	IM
15	15A	KIT exon 11 deletion Y553-K558; exon 17 N822 K	Male	42	Stomach	Metastasis	Y	IM
16	16A	KIT exon 11 deletion W557-K558; exon 17 D816G	Male	45	Small intestine	Metastasis	Y	IM
	16B	KIT exon 11 deletion W557-K558; exon 17 Y823D	Male	45	Small intestine	Metastasis	Y	IM
17	17A	KIT exon 11 deletion 551_554PMYE > Q	Male	65	Small intestine	Primary tumor	Y	None
18	18A	KIT exon 9 insertion AY502–503	Female	41	Small intestine	Primary tumor	Y	None
19	19A	KIT exon 11 V559D	Female	65	Stomach	Metastasis	Y	None
20	20A	KIT exon 13 K642E	Female	62	Small intestine	Primary tumor	N	None
21	21A	KIT exon 11 deletion W557-K558	Male	74	Stomach	Metastasis	Y	IM

*DA* dasatinib, *DOX* doxorubicin, *GEM* gemcitabine, *IM* imatinib, *LBH* LBH-589, panbinostat, *NI* nilotinib, *PA* pazopanib, *RAP* rapamycin, *RE* regorafenib, *SU* sunitinib, *SO* sorafenib, *Y/N* (yes/no)

### Whole exome sequencing

The average whole exome sequencing read depth for each tumor sample was 88X (75X median) (Additional file 1: Figure S1). Only 4 of the 21 patients had samples with coverage less than 50×, with a 40–49× average (41–48× median) coverage over the targeted panel. Given the high purity of GIST samples (80–90% tumor cells), coverage was deemed adequate for identifying somatic variants of interest and only mutations with a read depth of at least 20 reads were included in our analysis. Across the entire cohort of GIST specimens, we identified a total of 4303 exonic or splice-site, non-synonymous somatic mutations including 1278 missense, 123 nonsense, 82 splice site, and 4 read-through mutations. In addition, we identified 157 deletion and 35 insertion mutations (Additional file 1: Table S1) [18].

It is well established that detection of larger insertion/deletion mutations (“indels”) by next-generation sequencing is challenging. Therefore, we used Sanger sequencing to identify or verify primary *KIT* insertion/deletion mutations, especially for *KIT* exon 9 and 11 mutations. Known primary insertion/deletion mutations not called by our indel detector were verified by visual inspection of whole exome sequencing reads.

### GIST mutational signature

**Fig. 1:**
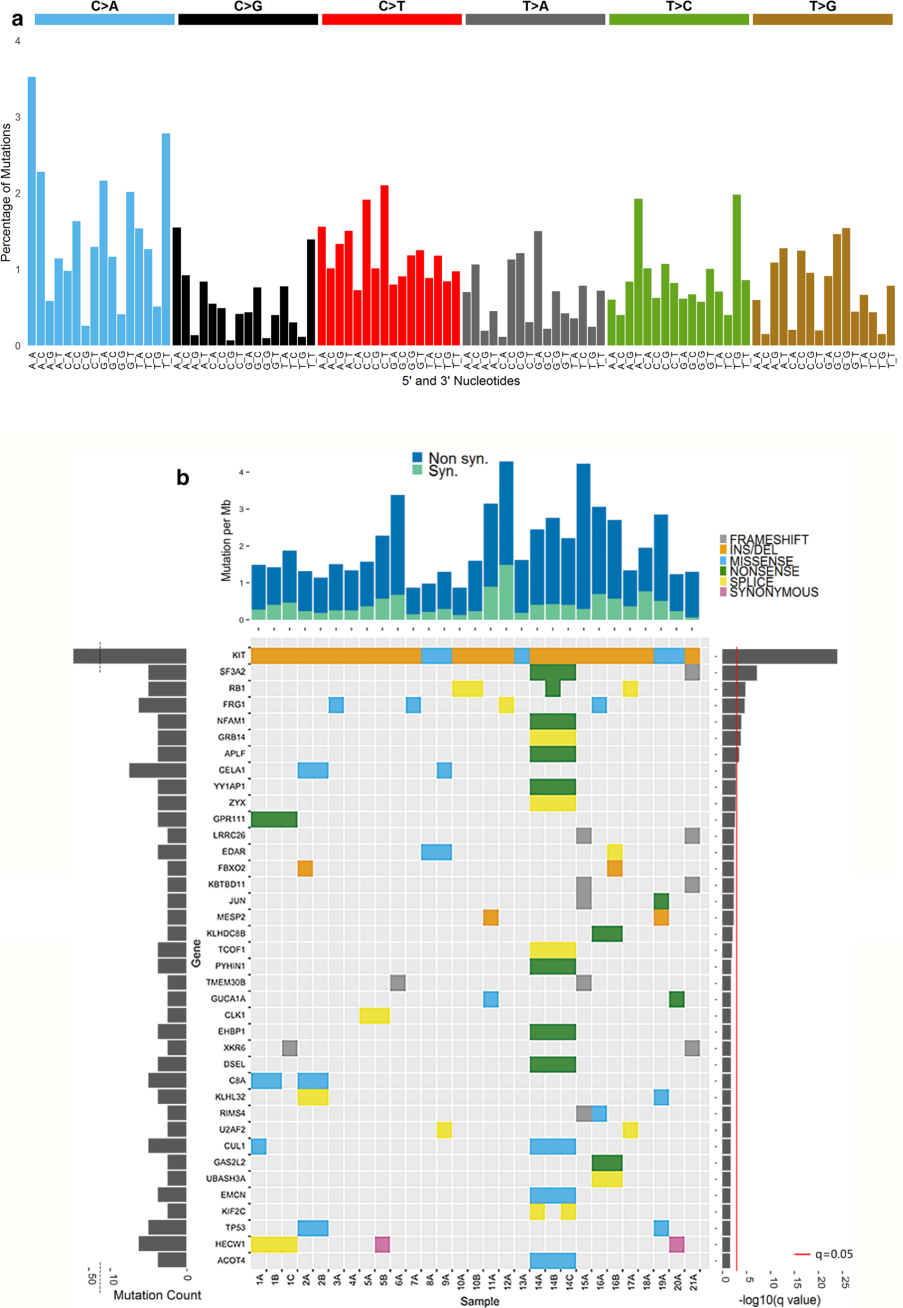
Mutational signature of 29 KIT-mutant GIST. **a** Average sequence context of somatic coding mutations for 40 GIST samples from WES. Signature displayed is according to the 96-substitution classification defined by the flanking 5′ and 3′ sequence adjacent to the mutated base. The mutation types are on the horizontal axis, vertical axis is the percentage of mutations attributed to each mutation type. Mutation signatures were normalized to the trinucleotide frequency of whole human genome. **b** Significantly mutated genes with q-value < 0.2 (corresponding p-value 0.0003) in KIT mutant GISTs. Main panel, variant type of significantly mutated genes found in each sample of our cohort of samples. Left panel represents the number of mutations of each gene. Right panel indicates the negative log_10_ of the q-value. Top panel represents the mutations per megabase (Mb) of the total coverage for each sample

An average of 122 somatic mutations were identified in the 29 *KIT*-mutant GIST samples (Additional file 1: Table S1). Genetic significance was determined based on the frequency of somatic variants with MutSigCV 1.4, which accounts for heterogeneity in mutational rate across the genome [19]. The mutation pattern of *KIT*-mutant GISTs consists largely of CpG transversions to A/T mutations, occurring at a relative rate of approximately 3.3 mutations per megabase (Mb) (Additional file 1: Table S2) [20]. The mutation signature was assessed by incorporating the sequence context of bases immediately 5′ and 3′ to the mutated base [21]. Percentages of mutations were normalized using the trinucleotide frequency in the human exome to the genome, leading to a decrease of C > T mutations occurring in NpCpG trinucleotides from 3 to 4% to 2% (Fig. 1a). The normalized signature show similarities to several COSMIC Signatures of Mutational Processes in Human Cancer, including Signature 5 (transcriptional strand bias for T > C substitutions at ApTpN context) and Signature 18 (neuroblastoma, breast and stomach carcinoma) [22]. The high frequencies of transition mutations at CpG in GIST are consistent with results of mutation rates of other gastrointestinal malignancies, including colorectal carcinoma, as previously reported [19]. Overall, the tumor mutation burden in these tumors was low (Additional file 1: Table S2), a finding with implications for the development of immunotherapy for GIST [23]. Ranked by q-value (lowest q-value first), the ten most commonly mutated genes in our collection of *KIT*-mutant GIST were *KIT, SF3A2, RB1, FRG1, NFAM1, GRB14, APLF, CELA1, YY1AP1,* and *ZYX* (Fig. 1b).

### Somatic copy number alterations

Various recurrent cytogenetic events have been described in GIST including deletions of chromosomes 14q, 22q, 15q, 1p, 9p and gains of chromosome 8q [24–28]. Large copy number events affecting more than 20% of a chromosomal arm were computationally identified using sequencing data (Table 2, Additional file 1: Table S3). Consistent with the above-cited literature, 23 out of 29 samples (16/21 patients) exhibited heterozygous loss of the long arm of chromosome 22q, and 24 of 29 samples (17/21 patients) had heterozygous copy number loss of the long arm of chromosome 14q (encompassing *RBM25*, *HSP90AA1*, and *MAX*).

**Table 2:** Regions of large-scale, chromosomal copy number variation in 29 KIT-mutant GIST (discovery set)

Genomic location	Event	Number of patients 21 total patients	Number of samples with CNV 29 total samples	Number of Genes with somatic SNV within CNV	Mean fraction of chromosomal arm	Fraction GIST samples with CNV	SNV mutated Genes from Cell Cycle (and others) pathways found in regions of large scale CNV. Bolded gene names indicated genes mutated in more than one sample
14q	LOSS	16	25	24	0.929948779	0.862068966	ACOT4, ADAM20, BCL11B, COCH, DTD2, DYNC1H1, FLVCR2, **HSP90AA1**, KCNH5, LTBP2, MYH7, NGB, OR4M1, OR4N2, PSMB11, **RBM25**, SERPINA1, SERPINA9, TMEM30B
1p	LOSS	16	25	43	0.838940558	0.862068966	ABCA4, ALDH4A1, B4GALT2, C8A, CCDC24, CD2, CDCP2, CROCC, CSDE1, CYP2J2, ELTD1, FBXO2, **GADD45A**, GJB5, GPR153, GRIK3, HSPG2, IGSF3, **JUN**, KIF2C, NBPF1, PALMD, RAP1GAP, RBMXL1, RERE, **SF3A3**, SRRM1, SZT2, TNFRSF8, TRIM45, TTC39A, WRAP73, ZMYM4, ZNF326
22q	LOSS	17	23	17	0.878011687	0.793103448	**BCR**, CHADL, GTPBP1, HDAC10, LZTR1, MLC1, NFAM1, NUP50, PEX26, RBFOX2, SOX10, TTC28
15q	LOSS	13	21	29	0.940300484	0.724137931	ANKRD34C, CHRNA5, CSPG4, FAM169B, **IGF1R**, IQGAP1, MAN2A2, MAP1A, MESP2, MGA, MORF4L1, MYO5C, NEO1, RFX7, RMDN3, RNF111, RYR3, SKOR1, SPINT1, SPTBN5, STARD5, TBC1D2B, TCF12, TJP1, TP53BP1, USP3
13q	LOSS	12	20	13	0.888207393	0.689655172	DACH1, LACC1, MYCBP2, PARP4, **RB1**, SHISA2, TPP2, ZC3H13
5p	GAIN	10	14	10	0.928171594	0.482758621	ADAMTS16, CDH10, CDH6, DAB2, OXCT1, PLEKHG4B, PRDM9
5q	GAIN	9	9	25	0.924167057	0.310344828	ACSL6, AGGF1, ARSI, **CCNB1**, CLINT1, **DDX46**, EBF1, FAM71B, FAT2, GNB2L1, **MSH3**, NDUFAF2, PCDHA1, PCDHB16, PCDHB7, PCDHGB6, PCSK1, RASGRF2, RBM27, TCOF1, TMCO6
7q	GAIN	6	8	17	0.74250744	0.275862069	CCDC136, CEP41, **CUL1**, FKBP6, **MET**, NOM1, OPN1SW, PCLO, PON1, TMEM168, TMEM176A, TRPV5, ZNF425, ZNF727
8q	GAIN	7	8	11	0.818582114	0.275862069	AGO2, COL14A1, EPPK1, NCOA2, PLEC, TNFRSF11B, TRPA1, ZFPM2

Additional heterozygous large chromosomal copy number losses were detected for a large region of chromosome 1p (encompassing *SF3A3*, *RBMXL1*, and *MAD2L2*) in 25/29 tumors (18/21 patients), chromosome 15q in 21/29 tumors (13/21 patients), and chromosome 13q (including *RB1*) in 20/29 tumors (11/21 patients), respectively. These results are consistent with prior studies [29–31].

KIT-mutant GISTs had heterozygous copy number gains affecting more than 20% of chromosomes arms 5p (14/29 tumors, 10/21 patients), 8q (10/29 tumors, 7/21 patients), and 7q (8/29 tumors, 6/21 patients) [31]. Heterozygous copy number gains affecting chromosome 5q (including *CCNB1*, *DDX46*) were observed in 8/29 GIST samples (6/21 patients; Table 2).

### Enrichment for Somatic Mutation of Cell Cycle Pathway-Related Genes in GISTs

Pathway analysis using the list of genes associated with exonic/splice site, non-silent somatic mutations in the *KIT*-mutant GISTs was performed using KEGGREST v1.8.1. This analysis not only showed significant enrichment in cancer-related pathways and other *KIT*-associated pathways, but also enrichment for the KEGG cell cycle pathway (hsa04110; Fisher’s Exact p = 0.001, Additional file 2: Table S4) [32]. In addition, gene set enrichment analysis (GSEA) of somatic mutations using MutSigCV against a gene enrichment database, Molecular Signatures Database (MsigDB) v5.1, showed enrichment of genes associated with the cell cycle pathway (Reactome, M543, p = 0.011, Additional file 3: Table S5).

**Fig. 2:**
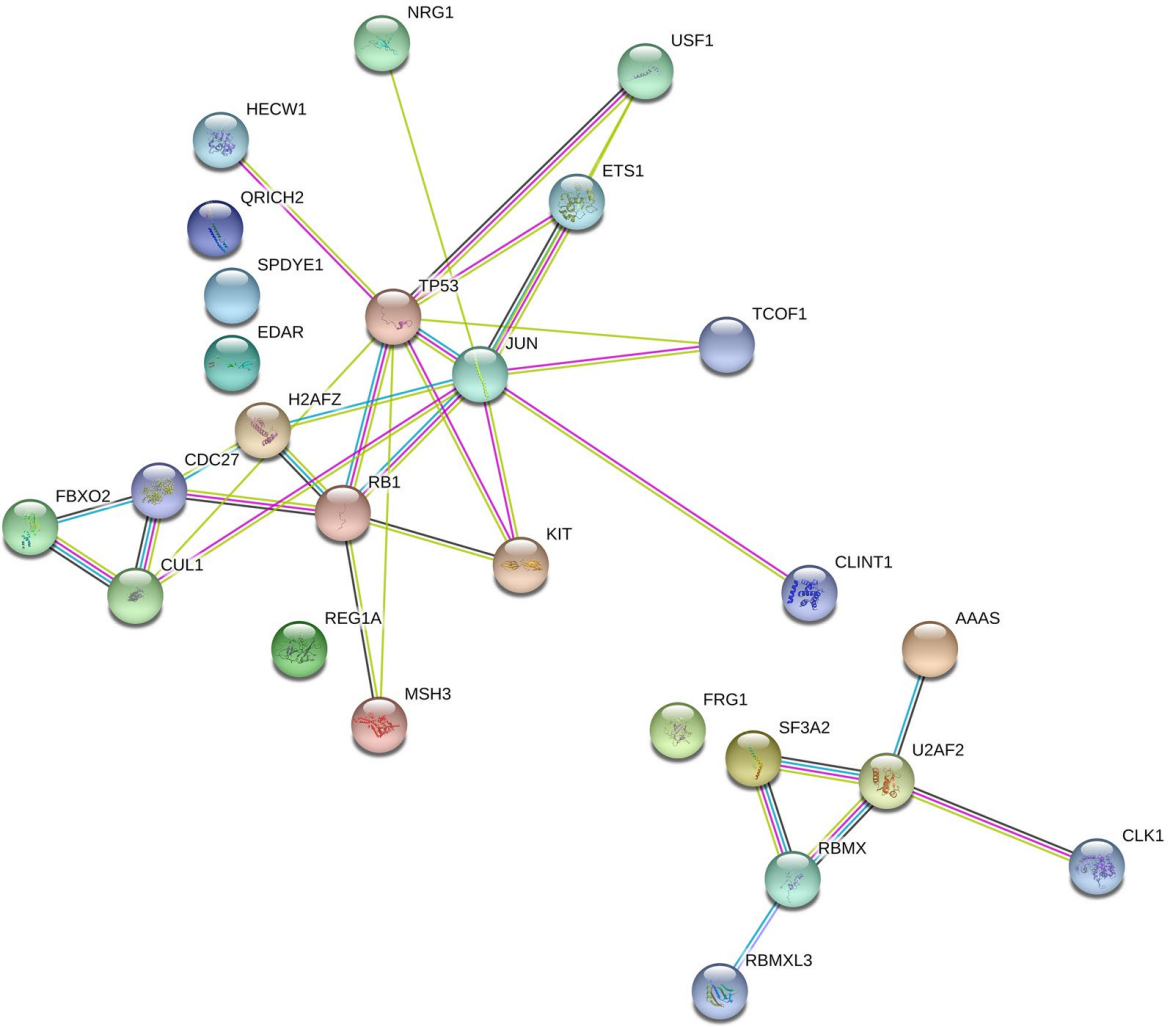
KIT mutant GISTs have enrichment for KEGG cell cycle pathway (p = 0.0007) and spliceosome pathway (p = 0.0008) using STRING v10 clustering of significantly somatic mutated genes (p < 0.003). The left cluster represents the cell cycle genes *CDC27*, *CUL1*, *TP53*, *RB1* and closely related mutated genes. The cluster on the right represents genes involved in spliceosome regulation, *SF3A2*, *U2AF2*, *RBMX* and other closely related genes

A total of ten KEGG cell cycle-associated genes were found to be significantly mutated by MutSigCV (nominal p < 0.05) in the entire cohort of GIST patients (Additional file 4: Tables S6, Additional file 5: Tables S7) [19]. These mutated cell cycle-related genes had 14 unique somatic exonic or splice site-related non-synonymous point mutation events that were filtered by variant allelic fraction greater than 0.2 to eliminate potential false positive results due to the presence of pseudogenes. The significantly mutated cell cycle genes in our cohort of *KIT*-mutant GISTs consisted of eight genes: *RB1* (p = 1.3e−6), *CUL1* (p = 3.1e−4), *TP53* (p = 3.7e−4), *CDC27* (p = 2.0e−3), *ANAPC1* (7.4e−3), *E2F3* (2.2e−2), *GADD45A* (p = 4.9e−2), and *MAD2L2* (p = 0.05) (Table 3). Evaluation using STRINGv10 of significantly mutated genes showed significant enrichment for the cell cycle pathway (p = 0.0007) and spliceosome-related genes (p = 0.0008) in *KIT*-mutant GISTs (Fig. 2) [33].

**Table 3:** Significant exonic or splice site non-synonymous mutations of KEGG cell cycle genes by MutSigCV in *KIT* mutant GIST samples

Hugo symbol	Number of patients	Samples	Variant classification	Variant type	Genome_change	Exon	Codon change	Protein change	Gene p value	Mean VAF	Range VAF
RB1	1	17A	Splice_Site	DEL	g.chr13:48953728_48953729delAG	14	c.e14-1		1.31E−06	0.50	0.50
RB1	1	10A, 10B	Splice_Site	SNV	g.chr13:49030485G > A	19	c.(1960–1962)Gtg > Atg	p.V654 M	1.31E−06	0.52	0.18–0.86
RB1	1	14B	Nonsense_Mutation	SNV	g.chr13:48954195G > T	15	c.(1396–1398)Gaa > Taa	p.E466*	1.31E−06	1.00	1
CUL1	1	14A, 14B, 14C	Missense_Mutation	SNV	g.chr7:148451142C > T	3	c.(214–216)tCg > tTg	p.S72L	3.11E−04	0.54	0.41–0.54
TP53	1	2A, 2B	Missense_Mutation	SNV	g.chr17:7577548C > T	7	c.(733–735)Ggc > Agc	p.G245S	3.66E−04	0.92	0.89–0.92
TP53	1	20A	Missense_Mutation	SNV	g.chr17:7573984A > G	10	c.(1042–1044)tTg > tCg	p.L348S	3.66E−04	0.50	0.5
TP53	1	20A	Missense_Mutation	SNV	g.chr17:7577548C > A	7	c.(733–735)Ggc > Tgc	p.G245C	3.66E−04	0.41	0.41
CDC27	1	8A	Missense_Mutation	SNV	g.chr17:45266534G > A	1	c.(4–6)aCg > aTg	p.T2 M	2.00E−03	0.33	0.33
ANAPC1	1	15A	Frame_Shift_Del	DEL	g.chr2:112622408_112622409delCA	8	c.(805–807)tggfs	p.W269 fs	7.36E−03	0.50	0.5
E2F3	1	10A, 10B	Missense_Mutation	SNV	g.chr6:20486985T > C	5	c.(949–951)gTt > gCt	p.V317A	2.23E−02	0.34	0.19–0.49
GADD45A	1	21A	Missense_Mutation	SNV	g.chr1:68153434G > A	4	c.(475–477)Gtg > Atg	p.V159 M	4.88E−02	0.42	0.42
MAD2L2	1	15A	Missense_Mutation	SNV	g.chr1:11735761G > C	9	c.(454–456)aCg > aGg	p.T152R	4.97E−02	0.77	0.77

### Integrated mutation and copy number analysis of cell cycle genes

**Fig. 3:**
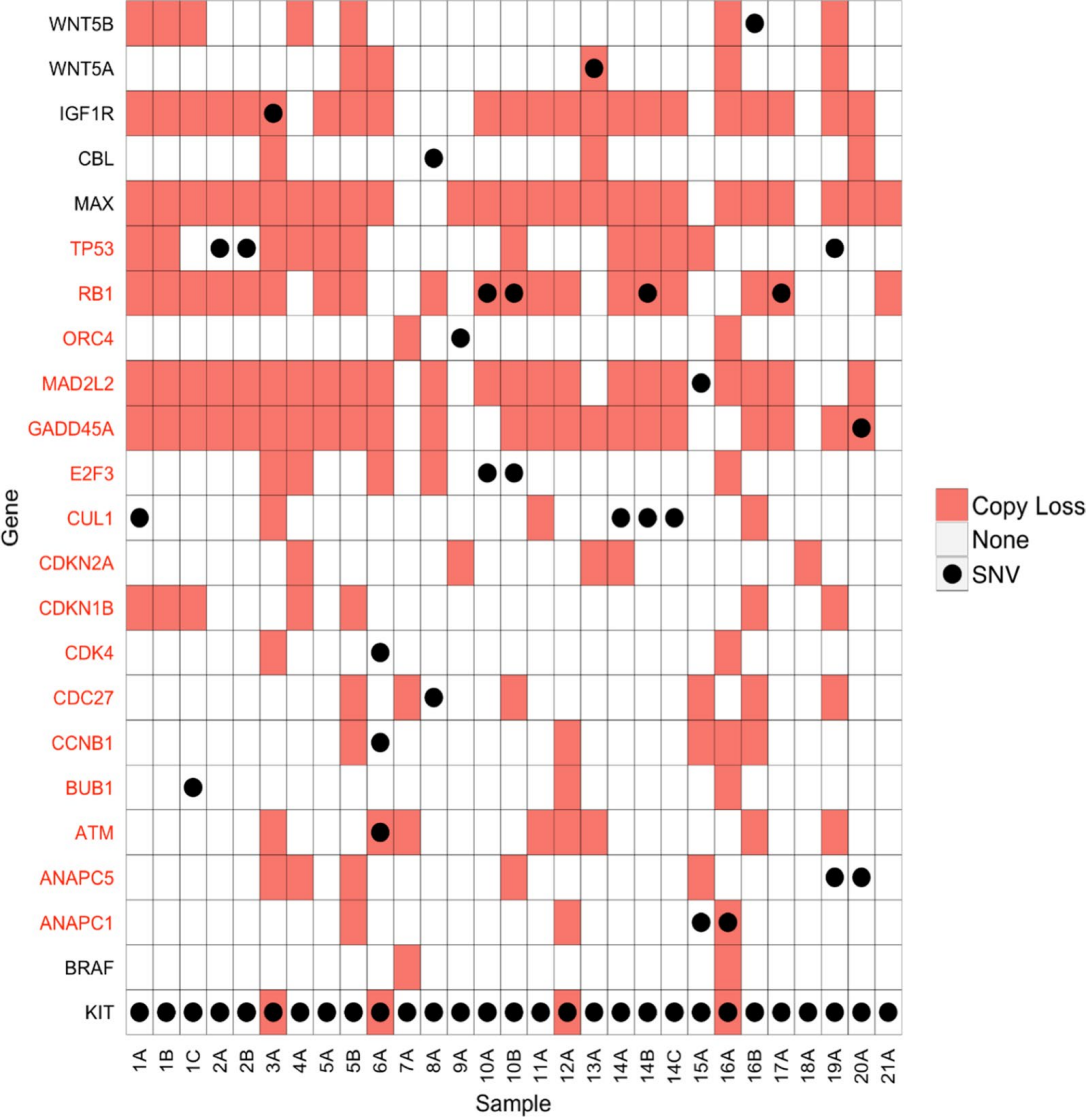
Integrated genomic analysis of somatic mutation and copy number variations of KEGG pathway cell cycle genes in *KIT*-mutant GIST. The integrated results from genomic copy number loss and somatic mutation analyses are shown. Genes with a deleterious single nucleotide variant* (●) in a gene within the cell cycle pathway (red font on left) or cancer pathways (black font on left) are shown with the corresponding copy number variation for the same gene (red boxes). *Exonic, non-synonymous or splicing

To delineate the spectrum of molecular mechanisms that inactivate cell cycle genes in advanced GIST, we integrated the data from the somatic variant analysis with the inferred copy number variation data from the sequencing analysis. As shown in Fig. 3, *TP53 (*9/21 patients, 14/29 tumors), *RB1* (12/21 cases, 19/29 tumors), *CDKN2A* (5/22 cases, 5/29 tumors), and *MAX* (17/21 patients, 25/29 tumors) were frequently altered by somatic mutation and/or copy number variation. Hemizyous/homozygous inactivating single nucleotide mutations of MAX were previously reported in 8/76 (10.5%) of GIST [34]. We did not any identify any inactivating MAX mutations in our series; this difference may be due to our smaller sample size. As noted above, large heterozygous deletions of chromosome 14 that included MAX were found in 17/21 (81%) patients in our WES cohort. Homozygous inactivation of MAX was previously reported to decrease expression of CDKN2A in GIST [34]. Based on our results, we speculate that haplo insufficiency of MAX may be enough to provide early dysregulation of the cell cycle (CDKN2A) during GIST pathogenesis.

### Somatic cell cycle mutations are conserved between individual tumors resected from the same patient

In general, any somatic cell cycle mutation found in one metastasis from a given patient was also found in the other metastases analyzed from that patient. For example, all three metastases analyzed of Patient 1 had the same *CDKN2A* homozygous deletion, as identified by Affymetrix array. Similarly, both metastases analyzed from Patient 2 had the same *TP53* c.(733–735) Ggc > Agc, p.G245S mutation at allelic fractions that were essentially homozygous (0.92 and 0.89, respectively; samples 2A and 2B, Table 3).

### Dystrophin

Deletion of *dystrophin* (*DMD*), a tumor suppressor gene in human cancers with a myogenic program, has been reported as a late genetic event associated with the development of metastatic potential in GIST [35]. In our cohort,* DMD* copy loss was observed in eleven of the 29 lesions of* KIT*-mutant GIST (8/21 patients). The majority of the* DMD* copy loss events occurred in exons 1 through 32 which impacts Dp427 myogenic dystrophin but preserves a ubiquitously-expressed Dp71 dystrophin, as reported previously [35]. Among the patients with multiple tumors sequenced (patients 1, 2, 10, 14, and 16), no DMD copy loss was found in samples from patients 10, 14, or 16. Samples from patient 1 (3 samples) and patient 2 (2 samples) had identical DMD deletions in all analyzed samples.

### Loss-of-heterozygosity (LOH) and targeted sequencing of CDKN2A, RB1 and TP53 in a cohort of 71 patients with well-defined GIST (validation data set)

**Fig. 4:**
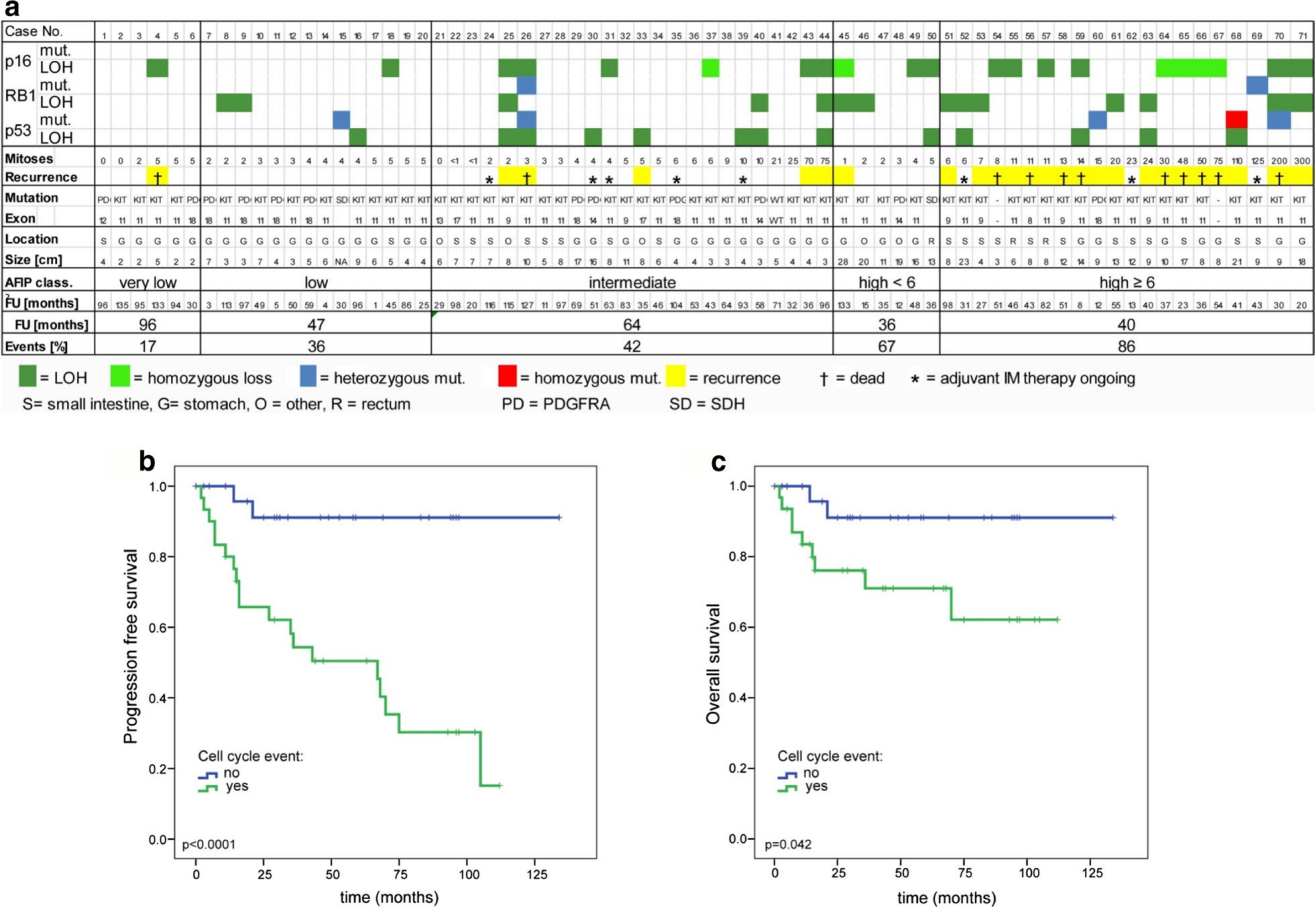
**a** Systematic screen for cell cycle-related genetic events in GIST with very low, low, intermediate and high risk of relapse according to the AFIP classification using Ion Torrent sequencing, MLPA and FISH analyses. Kaplan–Meier curves showing relapse-free (**b**) and overall survival (**c**) for patients with (green line) or without (blue line) a cell cycle-related event involving either *TP53*, *CDKN2A*, or *RB1*

Based on the whole exome sequencing results, we expanded our study to perform a focused genetic screen for mutations and/or LOH of the cell cycle regulators *CDKN2A/B*, *RB1* and *TP53* using a combination of a clinically validated next generation sequencing panel, MLPA, and FISH in a cohort of 71 GIST patients from well-defined clinical risk groups with annotated clinical follow up. *MAX* was excluded from these analyses as we viewed this as an early event that was unlikely to influence prognosis [34]. In addition to the AFIP (Armed Forces Institute of Pathology) criteria for risk of GIST recurrence, we further divided the high-risk group into patients with lower (< 6 mitoses/50 high power fields [HPF]) and those with higher mitotic counts (≥ 6 mitoses/50HPF). Genetic events (mutation, deletion, and/or LOH) involving at least one of the three genes examined (*CDKN2A/B*, *RB1, TP53*) were found in 17% (1/6) of the very low-risk, 36% (5/14) of the low-risk, 42% (10/24) of the intermediate risk, 67% (4/6) of the high-risk/low mitotic-count and in 86% (18/21) of the high-risk/high mitotic-count group (Fig. 4a). The presence of cell cycle-related events was associated with a significantly shorter relapse-free survival (median 67 months *versus* not reached; *p* < 0.0001; Fig. 4b) and overall survival (Log Rank, *p *= 0.042; Fig. 4C) in the univariate analysis. Patients who were receiving ongoing adjuvant imatinib treatment were excluded from our analyses. In a multivariate analysis, the proportional hazards assumption was fulfilled for all covariates, and Cox regression analysis revealed a hazard ratio of 3.9, 7.3 and 4.5 for cell cycle-related events, mitotic count, and tumor size, respectively (*p *= 0.0775; Additional file 6: Table S8).

## Discussion

The dependency of most GIST on oncogenic *KIT* mutations is indisputable. Consistent with this statement, our current whole exome sequencing studies revealed secondary *KIT* mutations in 18/29 tumors obtained from 21 patients with malignant *KIT*-mutant GIST (Table 1). Other recurrent molecular causes of TKI resistance such as acquired mutations of downstream signaling effectors (e.g., *KRAS* or *PIK3CA*) were not identified in our discovery sample set. We conclude that secondary *KIT* mutations are found in most cases of *KIT*-mutant GIST with acquired resistance to kinase inhibitors. These results are in agreement with prior sequencing studies that analyzed the frequency of secondary kinase mutations in TKI-resistant, *KIT*-mutant GIST [30, 36].

However, additional events besides primary *KIT* mutations are needed for GIST cells to evolve into metastatic or resistant disease, as evidenced by similar or identical *KIT* mutations in cases of micro-GISTs and metastatic tumors [25]. In this study, we provide evidence that mutational dysregulation of the cell cycle is a crucial mechanism in progression from low-risk to malignant GIST. Whole exome sequencing of a set of high grade or metastatic GIST specimens showed significant enrichment of mutations of cell cycle pathway-related genes (Fisher’s Exact p = 0.001). We found further evidence of genomic inactivation of cell cycle control genes when analyzing copy number loss using the whole exome sequencing data. Overall, the most common recurrently copy number loss events were found in *MAX* (17/21 patients) and the cell cycle-related genes *RB1* (13/22 patients), *TP53* (12/21 patients), *CDKN1B* (5/21 cases), and *CDKN2A/B* (5/21 cases). Samples with somatic mutations of cell cycle genes and/or copy number losses of these same genes are summarized in Fig. 3.

Because the whole exome sequencing results in our present series underscored the importance of genomic inactivation of cell cycle regulator genes in GIST, we performed focused testing of the genomic status (mutation, copy number variation, and LOH) of three of these genes (*CDKN2A*, *RB1*, and *TP53*) in an independent group of GIST patients with annotated long-term clinical follow up (validation data set). We found that mutations and/or copy loss of these cell cycle regulator genes were associated with an increased risk of metastatic recurrence. In this second group of patients, we noted a significantly lower percentage of *TP53, CDKN2A/B* or *RB1* genomic alterations in low-risk compared to high-risk GIST (Fig. 4). All but two of the patients who later experienced recurrence had at least one genomic alteration involving *CDKN2A/B*, *RB1*, or *TP53*. Notably, cell cycle-related genetic events were associated with higher number of mitoses in primary GISTs (Fig. 4a), a well-recognized risk factor for the development of metastatic disease.

Patients with intermediate-risk GISTs are clinically challenging, because there are currently no reliable biomarkers to identify the patients in this risk category who have a higher risk of relapse and might benefit from adjuvant imatinib therapy. Among the 24 intermediate-risk GISTs in our second cohort, ten (42%) had cell cycle-related genetic events. Notably, among these ten patients, seven did not receive adjuvant imatinib and five patients experienced a recurrence of their GIST. By contrast, none of the 14 patients with intermediate-risk GIST lacking a genomic cell cycle event had a recurrence (2-sample proportion test, p = 0.002). Overall, a cell cycle-related genetic event was strongly associated with poor progression-free survival in patients with GIST (*p *< 0.0001). Notably, the only patient with metastatic relapse in the very low-risk group of six patients had LOH of the *CDKN2A/B* locus in his gastric primary tumor.

In a multivariate analysis, both mitotic count and tumor size were highly significant prognostic factors. Due to the limitations of sample size, primary tumor site was excluded from the analysis. Nonetheless, multivariate analysis for recurrence-free survival revealed a hazard ratio of 3.9 for GISTs with a cell cycle-related genetic event (*p *= 0.078) even against the background of a relatively small group of patients. Further assessment of the clinical validity of these genetic events as prognostic factors will require larger numbers of patients with annotated long-term clinical follow-up. Our results are generally concordant with other studies that correlated cell cycle events and risk of GIST recurrence. However, these studies used a variety of techniques, including immunohistochemistry, to identify cell cycle gene dysregulation [37–40]. As shown in our studies, analysis of both specific cell cycle gene mutation status and CNV are necessary to identify a more complete spectrum of genomic events targeting RB1, CDN2A/B, and TP53 genes. Our experimental methods may not detect all intragenic deletions (e.g. microdeletions). In addition, in some cases of malignant GIST, CDKN2A is inactivated by promoter hypermethylation [41, 42]. Further studies are needed to identify the optimal diagnostic techniques needed to identify all mechanisms of inactivation of cell cycle genes in GIST. If validated in subsequent studies, these cell cycle markers could be used for prognostication in conjunction with standard pathological features that are known to correlate with risk.

In addition to the recurrent genomic events targeting cell cycle control genes, we also discovered evidence for dysregulation of mRNA splicing in the biology of metastatic GIST. Gene set enrichment analysis found *KIT*-mutant GISTs to also be enriched for mutations of genes involved in spliceosome regulation (*p* value = 0.026), including *SF3A2* (q < 0.001), *U2AF2*, *RBMX*, *RBMXL3*, *PRPF18*, *SNRP200* that were significantly mutated in *KIT*-mutant GISTs with p < 0.05. *SF3A2* and *U2AF2* are both associated with spliceosome component U2 small nuclear ribonucleotide proteins (snRNP) [43]. Three lesions from a single patient (samples 14A, 14B, 14C) contained the identical *SF3A2* nonsense mutation E177*. Two other tumors from different patients (9A and 17A) contained identical somatic *U2AF2* splice site mutations. The remaining mutated genes involved in spliceosome assembly with p < 0.001 were identified in nine other samples from eight different patients. In addition, four *KIT*-mutant samples contained somatic single nucleotide variants in *FRG1* (q < 0.01), a cancer driver gene in follicular thyroid cancer [44].

Notably, mutations of *SF3A2* and *FRG1* ranked as the first and third most common somatic mutations (after *KIT*), respectively in *KIT*-mutant GIST (Fig. 1b). SF3A2 is part of the U2 snRNP, and another member of this complex (SF3B1) is recurrently mutated in myelodysplastic syndrome, acute myeloid leukemia, and chronic lymphocytic leukemia [45]. We also identified loss of the SF3A3 gene, located on 1p, to be a frequent event in our series (17/21 patients, 25/29 samples). Like SF3A2, the SF3A3 gene encodes a component of the splicing factor 3a complex. In addition, we found mutations of U2AF2, a component of the U2AF complex. U2AF2 mutations leading to spliceosome dysfunction have been reported in other cancers [46]. Notably, mutation of the other member of the U2AF complex (U2AF1) has been reported in the same hematologic malignancies associated with SF3B1 mutations [47]. Further studies are needed to understand how dysregulation of the spliceosome contributes to GIST biology.

**Fig. 5:**
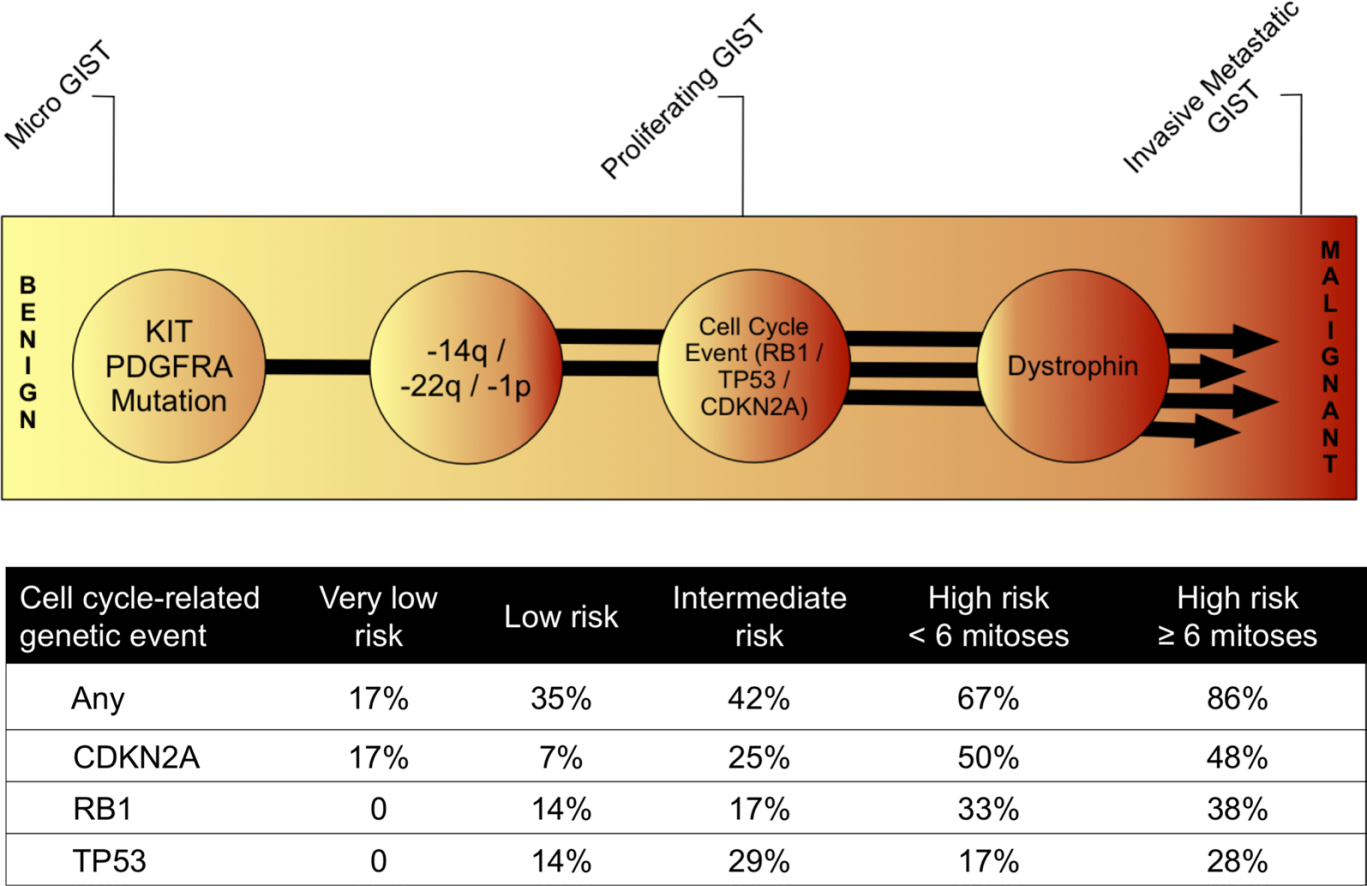
Model of genetic progression of GIST from benign to invasive/metastatic tumors

Taken together, our study provides evidence that mutational dysregulation of the cell cycle is a crucial mechanism in progression from low-risk to malignant GIST. Based on these observations we propose a model of genetic progression leading to malignant, invasive and metastatic GIST as shown in Fig. 5. Our model depicts chromosome 14q and 22q deletions as early events in GIST progression. *KIT*-mutant asymptomatic and innocuous micro-GISTs have been found in approximately one-third of the general population. These tumors rarely progress to malignancy, presumably because they require additional mutations to become clinically aggressive. Cytogenetic studies support this hypothesis, demonstrating that early GISTs typically harbor *KIT* or *PDGFRA* mutations, often accompanied by chromosome 14q and 22q deletions, but do not yet show evidence of chromosome 9p deletions that potentially target *CDKN2A/B* [26]. In contrast, inactivation of dystrophin is a late event in GIST progression (Fig. 5). Dystrophin has been shown to behave as a tumor suppressor, and in a large series of GISTs genomic mechanisms of dystrophin inactivation were present in 96% of metastatic GIST, but absent in low-risk GIST [36]. We therefore propose that cell cycle-related events are ubiquitous, intermediate steps in the progression of GIST. Our observations also suggest that clinical assessment of genetic events involving *CDKN2A/B*, *RB1* or *TP53* may be a useful addition to existing risk assessment models, with implications for selection of patients for adjuvant treatment and intensified surveillance schedules.

## Conclusions

Our studies show that cell cycle dysregulation due to genomic inactivation of cell cycle regulatory genes is a ubiquitous mechanism during the transition from low-risk to high-risk/metastatic GIST. These events might sensitize cells to KIT-independent treatment approaches such as CDK4 or MDM2 inhibitors. Based on this data, we propose a model for molecular pathogenesis of malignant GIST. Further studies are needed to confirm these genomic observations as potential predictors of clinical behavior and to examine the efficacy of imatinib combination therapy with cell cycle inhibitors in GIST. In addition, further studies are needed to understand the role of spliceosome mutations in malignant GIST biology.

## Additional files

**Additional file 1.** Additional methods for genomic studies. **Figure S1.** Comparison of WES read depth (blue bars) and SNV frequency (orange bars). **Table S1.** Mutation type frequency in 29 *KIT*-mutant GIST. **Table S2.** Breakdown of mutation rates per category discovered for this individual set. **Table S3.** Regions of large copy number variations affecting greater than 20% of the bases of a given chromosome arm. The numbers represent proportion of bases in the specified chromosomal arm that exhibit copy number variation.

**Additional file 2: Table S4.** KEGG pathways enrichment analysis results.

**Additional file 3: Table S5.** Gene set enrichment analyses for significant mutations according to Mutsig assessment.

**Additional file 4: Table S6.** MutSig analysis results. All mutations-sample codes match manuscript numbering.

**Additional file 5: Table S7.** All somatic mutations. Sample codes match manuscript numbering (e.g. 1A, 1B, etc.).

**Additional file 6: Table S8.** Multi-variate analysis of clinically characterized GIST.

## References

[1] Hirota S, Isozaki K, Moriyama Y, Hashimoto K, Nishida T, Ishiguro S (1998). Gain-of-function mutations of c-kit in human gastrointestinal stromal tumors. Science.

[2] Heinrich MC, Corless CL, Demetri GD, Blanke CD, von Mehren M, Joensuu H (2003). Kinase mutations and imatinib response in patients with metastatic gastrointestinal stromal tumor. J Clin Oncol.

[3] Corless CL (2014). Gastrointestinal stromal tumors: what do we know now?. Mod Pathol.

[4] Liegl-Atzwanger B, Fletcher JA, Fletcher CDM (2010). Gastrointestinal stromal tumors. Virchows Arch Int J Pathol..

[5] Agaram NP, Wong GC, Guo T, Maki RG, Singer S, Dematteo RP (2008). Novel V600E BRAF mutations in imatinib-naive and imatinib-resistant gastrointestinal stromal tumors. Genes Chromosomes Cancer.

[6] Demetri GD, von Mehren M, Blanke CD, Van den Abbeele AD, Eisenberg B, Roberts PJ (2002). Efficacy and safety of imatinib mesylate in advanced gastrointestinal stromal tumors. N Engl J Med.

[7] Fanta PT, Sicklick JK, Betz BL, Peterson MR (2015). In vivo imatinib sensitivity in a patient with GI Stromal Tumor bearing a PDGFRA Deletion DIM842-844. J Clin Oncol.

[8] Blanke CD, Demetri GD, von Mehren M, Heinrich MC, Eisenberg B, Fletcher JA (2008). Long-term results from a randomized phase II trial of standard- versus higher-dose imatinib mesylate for patients with unresectable or metastatic gastrointestinal stromal tumors expressing KIT. J Clin Oncol Off J Am Soc Clin Oncol..

[9] Blay J-Y, Le Cesne A, Ray-Coquard I, Bui B, Duffaud F, Delbaldo C (2007). Prospective multicentric randomized phase III study of imatinib in patients with advanced gastrointestinal stromal tumors comparing interruption versus continuation of treatment beyond 1 year: the French Sarcoma Group. J Clin Oncol Off J Am Soc Clin Oncol..

[10] Demetri GD, van Oosterom AT, Garrett CR, Blackstein ME, Shah MH, Verweij J (2006). Efficacy and safety of sunitinib in patients with advanced gastrointestinal stromal tumour after failure of imatinib: a randomised controlled trial. Lancet Lond Engl..

[11] Demetri GD, Reichardt P, Kang Y-K, Blay J-Y, Rutkowski P, Gelderblom H (2013). Efficacy and safety of regorafenib for advanced gastrointestinal stromal tumours after failure of imatinib and sunitinib (GRID): an international, multicentre, randomised, placebo-controlled, phase 3 trial. Lancet Lond Engl..

[12] Heinrich MC, Marino-Enriquez A, Presnell A, Donsky RS, Griffith DJ, McKinley A (2012). Sorafenib inhibits many kinase mutations associated with drug-resistant gastrointestinal stromal tumors. Mol Cancer Ther.

[13] Agaimy A, Wünsch PH, Hofstaedter F, Blaszyk H, Rümmele P, Gaumann A (2007). Minute gastric sclerosing stromal tumors (GIST tumorlets) are common in adults and frequently show c-KIT mutations. Am J Surg Pathol.

[14] Corless CL, McGreevey L, Haley A, Town A, Heinrich MC (2002). KIT mutations are common in incidental gastrointestinal stromal tumors one centimeter or less in size. Am J Pathol.

[15] Kawanowa K, Sakuma Y, Sakurai S, Hishima T, Iwasaki Y, Saito K (2006). High incidence of microscopic gastrointestinal stromal tumors in the stomach. Hum Pathol.

[16] Muenst S, Thies S, Went P, Tornillo L, Bihl MP, Dirnhofer S (2011). Frequency, phenotype, and genotype of minute gastrointestinal stromal tumors in the stomach: an autopsy study. Hum Pathol.

[17] Call J, Walentas CD, Eickhoff JC, Scherzer N (2012). Survival of gastrointestinal stromal tumor patients in the imatinib era: life raft group observational registry. BMC Cancer..

[18] Van der Auwera GA, Carneiro MO, Hartl C, Poplin R, Del Angel G, Levy-Moonshine A (2013). From FastQ data to high confidence variant calls: the genome analysis toolkit best practices pipeline. Curr Protoc Bioinforma Ed Board Andreas Baxevanis Al..

[19] Lawrence MS, Stojanov P, Polak P, Kryukov GV, Cibulskis K, Sivachenko A (2013). Mutational heterogeneity in cancer and the search for new cancer-associated genes. Nature.

[20] Kandoth C, McLellan MD, Vandin F, Ye K, Niu B, Lu C (2013). Mutational landscape and significance across 12 major cancer types. Nature.

[21] Alexandrov LB, Nik-Zainal S, Wedge DC, Aparicio SAJR, Behjati S, Biankin AV (2013). Signatures of mutational processes in human cancer. Nature.

[22] COSMIC: Signatures of Mutational Processes in Human Cancer. http://cancer.sanger.ac.uk/cosmic/signatures. Accessed 25 July 2016.

[23] Rizvi NA, Hellmann MD, Snyder A, Kvistborg P, Makarov V, Havel JJ (2015). Cancer immunology. Mutational landscape determines sensitivity to PD-1 blockade in non-small cell lung cancer. Science..

[24] Gunawan B, von Heydebreck A, Sander B, Schulten H-J, Haller F, Langer C (2007). An oncogenetic tree model in gastrointestinal stromal tumours (GISTs) identifies different pathways of cytogenetic evolution with prognostic implications. J Pathol..

[25] Corless CL, Barnett CM, Heinrich MC (2011). Gastrointestinal stromal tumours: origin and molecular oncology. Nat Rev Cancer.

[26] Yang J, Du X, Lazar AJF, Pollock R, Hunt K, Chen K (2008). Genetic aberrations of gastrointestinal stromal tumors. Cancer.

[27] Ylipää A, Hunt KK, Yang J, Lazar AJF, Torres KE, Lev DC (2011). Integrative genomic characterization and a genomic staging system for gastrointestinal stromal tumors. Cancer.

[28] Heinrich MC, Rubin BP, Longley BJ, Fletcher JA (2002). Biology and genetic aspects of gastrointestinal stromal tumors: kIT activation and cytogenetic alterations. Hum Pathol.

[29] Heinrich MC, Corless CL, Duensing A, McGreevey L, Chen C-J, Joseph N (2003). PDGFRA activating mutations in gastrointestinal stromal tumors. Science.

[30] Schoppmann SF, Vinatzer U, Popitsch N, Mittlböck M, Liebmann-Reindl S, Jomrich G (2013). Novel clinically relevant genes in gastrointestinal stromal tumors identified by exome sequencing. Clin Cancer Res Off J Am Assoc Cancer Res..

[31] El-Rifai W, Sarlomo-Rikala M, Andersson LC, Knuutila S, Miettinen M (2000). DNA sequence copy number changes in gastrointestinal stromal tumors: tumor progression and prognostic significance. Cancer Res.

[32] KEGGREST. Bioconductor. http://bioconductor.org/packages/KEGGREST/. Accessed 12 Mar 2016.

[33] Szklarczyk D, Franceschini A, Wyder S, Forslund K, Heller D, Huerta-Cepas J (2015). STRING v10: protein-protein interaction networks, integrated over the tree of life. Nucleic Acids Res.

[34] Schaefer I-M, Wang Y, Liang C, Bahri N, Quattrone A, Doyle L (2017). MAX inactivation is an early event in GIST development that regulates p16 and cell proliferation. Nat Commun..

[35] Wang Y, Marino-Enriquez A, Bennett RR, Zhu M, Shen Y, Eilers G (2014). Dystrophin is a tumor suppressor in human cancers with myogenic programs. Nat Genet.

[36] Wang Y, Fletcher JA (2015). Cell cycle and dystrophin dysregulation in GIST. Cell Cycle.

[37] Lartigue L, Neuville A, Lagarde P, Brulard C, Rutkowski P, Tos PD (2015). Genomic index predicts clinical outcome of intermediate-risk gastrointestinal stromal tumours, providing a new inclusion criterion for imatinib adjuvant therapy. Eur J Cancer.

[38] Sabah M, Cummins R, Leader M, Kay E (2006). Altered expression of cell cycle regulatory proteins in gastrointestinal stromal tumors: markers with potential prognostic implications. Hum Pathol.

[39] Lagarde P, Pérot G, Kauffmann A, Brulard C, Dapremont V, Hostein I (2012). Mitotic checkpoints and chromosome instability are strong predictors of clinical outcome in gastrointestinal stromal tumors. Clin Cancer Res.

[40] Ihle MA, Huss S, Jeske W, Hartmann W, Merkelbach-Bruse S, Schildhaus H-U (2018). Expression of cell cycle regulators and frequency of TP53 mutations in high risk gastrointestinal stromal tumors prior to adjuvant imatinib treatment. PLoS ONE.

[41] House MG, Guo M, Efron DT, Lillemoe KD, Cameron JL, Syphard JE (2003). Tumor suppressor gene hypermethylation as a predictor of gastric stromal tumor behavior. J Gastrointest Surg Off J Soc Surg Aliment Tract..

[42] Okamoto Y, Sawaki A, Ito S, Nishida T, Takahashi T, Toyota M (2012). Aberrant DNA methylation associated with aggressiveness of gastrointestinal stromal tumour. Gut.

[43] Lin P-C, Xu R-M (2012). Structure and assembly of the SF3a splicing factor complex of U2 snRNP. EMBO J.

[44] Erinjeri NJ, Nicolson NG, Deyholos C, Korah R, Carling T (2018). Whole-exome sequencing identifies two discrete druggable signaling pathways in follicular thyroid cancer. J Am Coll Surg.

[45] Chesnais V, Kosmider O, Damm F, Itzykson R, Bernard OA, Solary E (2012). Spliceosome mutations in myelodysplastic syndromes and chronic myelomonocytic leukemia. Oncotarget..

[46] Glasser E, Agrawal AA, Jenkins JL, Kielkopf CL (2017). Cancer-associated mutations mapped on high-resolution structures of the U2AF2 RNA recognition motifs. Biochemistry.

[47] Ilagan JO, Ramakrishnan A, Hayes B, Murphy ME, Zebari AS, Bradley P (2015). U2AF1 mutations alter splice site recognition in hematological malignancies. Genome Res.

