# Supplementary material for: Genomic aberrations in cell cycle genes predict progression of KIT-mutant gastrointestinal stromal tumors (GISTs)
Source: Clin Sarcoma Res. 2019 Mar 5;9:3. doi: 10.1186/s13569-019-0112-7 (PMC6399846; doi:10.1186/s13569-019-0112-7)
Supplement: Supplementary file 1 — Additional file 1. Additional methods for genomic studies. Figure S1. Comparison of WES read depth (blue bars) and SNV frequency (orange bars). Table S1. Mutation type frequency in 29 KIT-mutant GIST. Table S2. Breakdown of mutation rates per category discovered for this individual set. Table S3. Regions of large copy number variations affecting greater than 20% of the bases of a given chromosome arm. The numbers represent proportion of bases in the specified chromosomal arm that exhibit copy number variation. [file 13569_2019_112_MOESM1_ESM.pptx]

## Slide 1
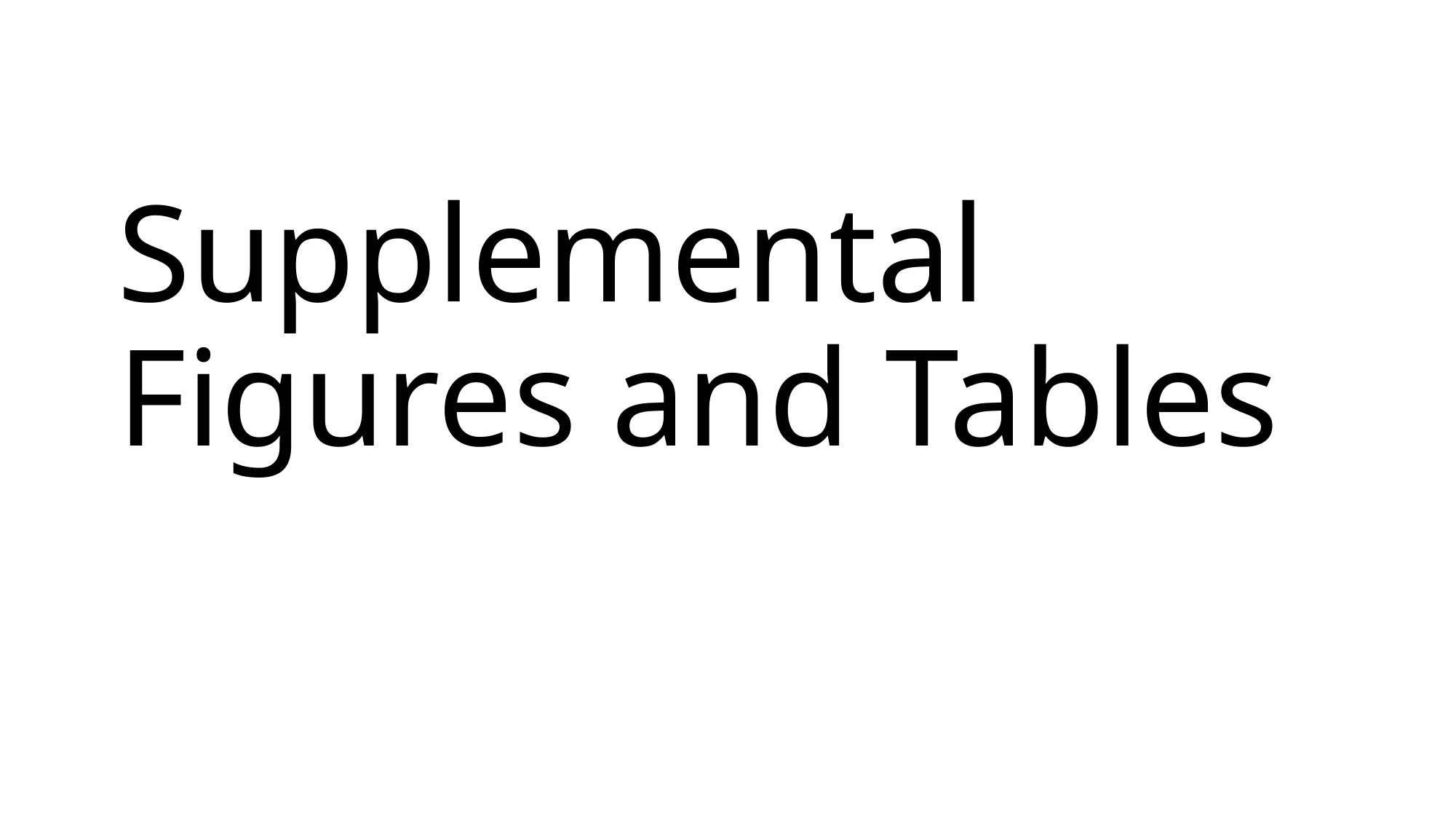

# Supplemental Figures and Tables

## Slide 2
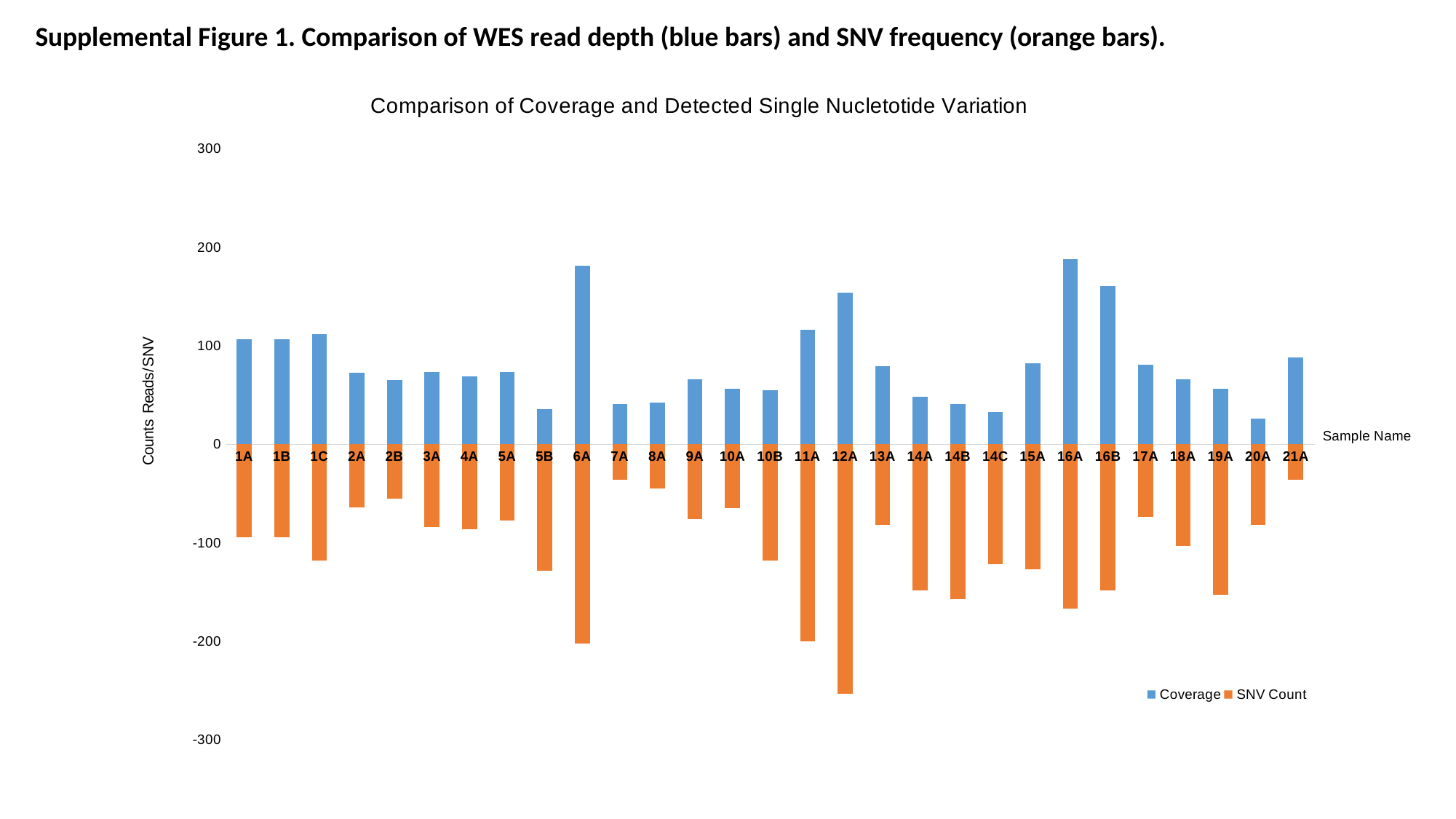

Supplemental Figure 1. Comparison of WES read depth (blue bars) and SNV frequency (orange bars).
### Chart: Comparison of Coverage and Detected Single Nucletotide Variation
| Category | Coverage | SNV Count |
|---|---|---|
| 1A | 106.6 | -94.0 |
| 1B | 106.55 | -94.0 |
| 1C | 111.56 | -118.0 |
| 2A | 72.45 | -64.0 |
| 2B | 65.54 | -55.0 |
| 3A | 73.26 | -84.0 |
| 4A | 69.23 | -86.0 |
| 5A | 73.23 | -77.0 |
| 5B | 36.02 | -128.0 |
| 6A | 181.26 | -202.0 |
| 7A | 41.04 | -36.0 |
| 8A | 42.31 | -45.0 |
| 9A | 66.41 | -76.0 |
| 10A | 56.74 | -65.0 |
| 10B | 55.17 | -118.0 |
| 11A | 116.64 | -200.0 |
| 12A | 154.36 | -253.0 |
| 13A | 79.35 | -82.0 |
| 14A | 48.29 | -148.0 |
| 14B | 41.17 | -157.0 |
| 14C | 33.19 | -122.0 |
| 15A | 82.13 | -127.0 |
| 16A | 187.65 | -167.0 |
| 16B | 160.34 | -148.0 |
| 17A | 80.84 | -74.0 |
| 18A | 66.21 | -103.0 |
| 19A | 56.34 | -153.0 |
| 20A | 26.35 | -82.0 |
| 21A | 88.14 | -36.0 |

## Slide 3
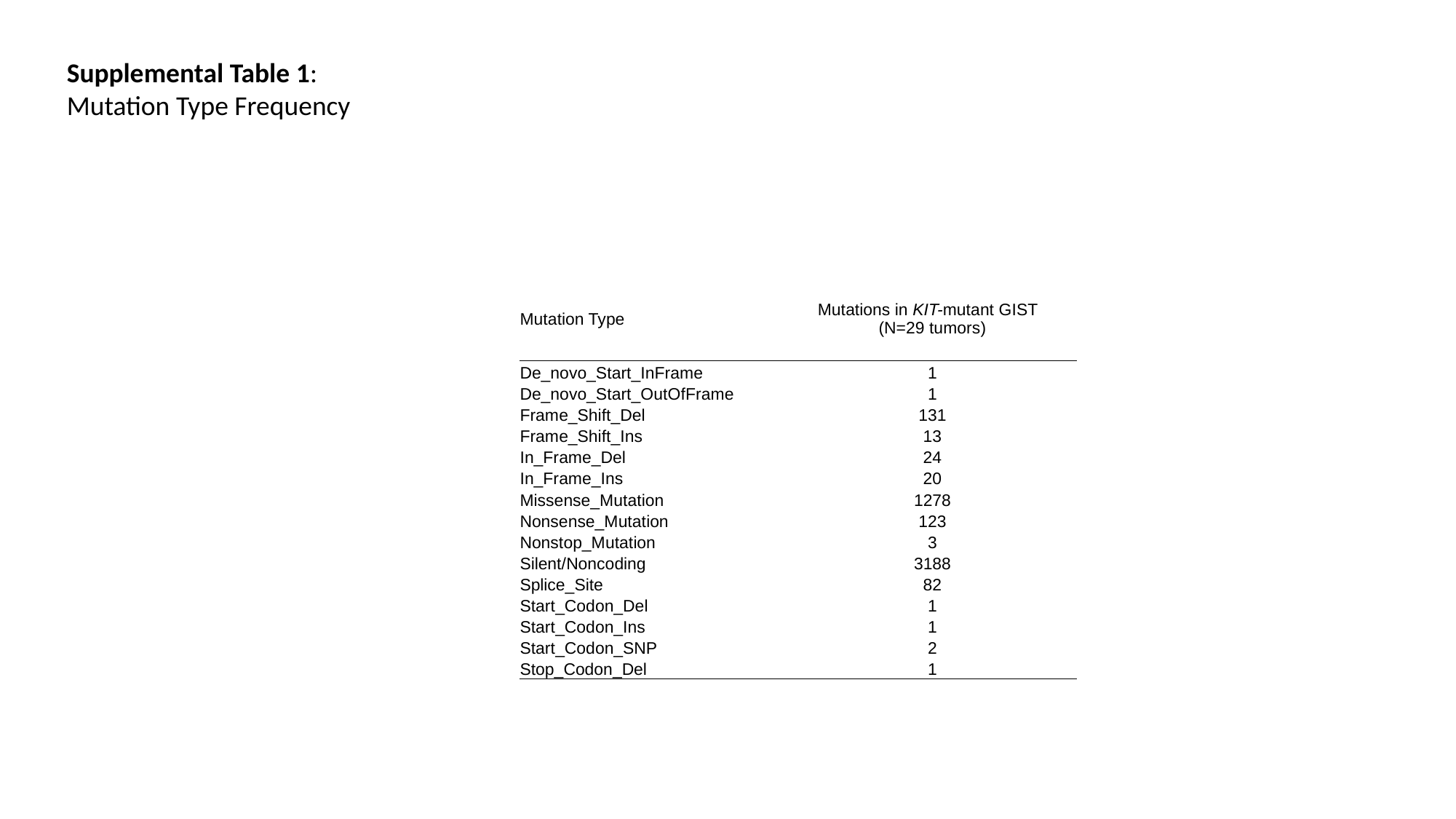

Supplemental Table 1: Mutation Type Frequency
| Mutation Type | Mutations in KIT-mutant GIST (N=29 tumors) |
| --- | --- |
| De\_novo\_Start\_InFrame | 1 |
| De\_novo\_Start\_OutOfFrame | 1 |
| Frame\_Shift\_Del | 131 |
| Frame\_Shift\_Ins | 13 |
| In\_Frame\_Del | 24 |
| In\_Frame\_Ins | 20 |
| Missense\_Mutation | 1278 |
| Nonsense\_Mutation | 123 |
| Nonstop\_Mutation | 3 |
| Silent/Noncoding | 3188 |
| Splice\_Site | 82 |
| Start\_Codon\_Del | 1 |
| Start\_Codon\_Ins | 1 |
| Start\_Codon\_SNP | 2 |
| Stop\_Codon\_Del | 1 |

## Slide 4
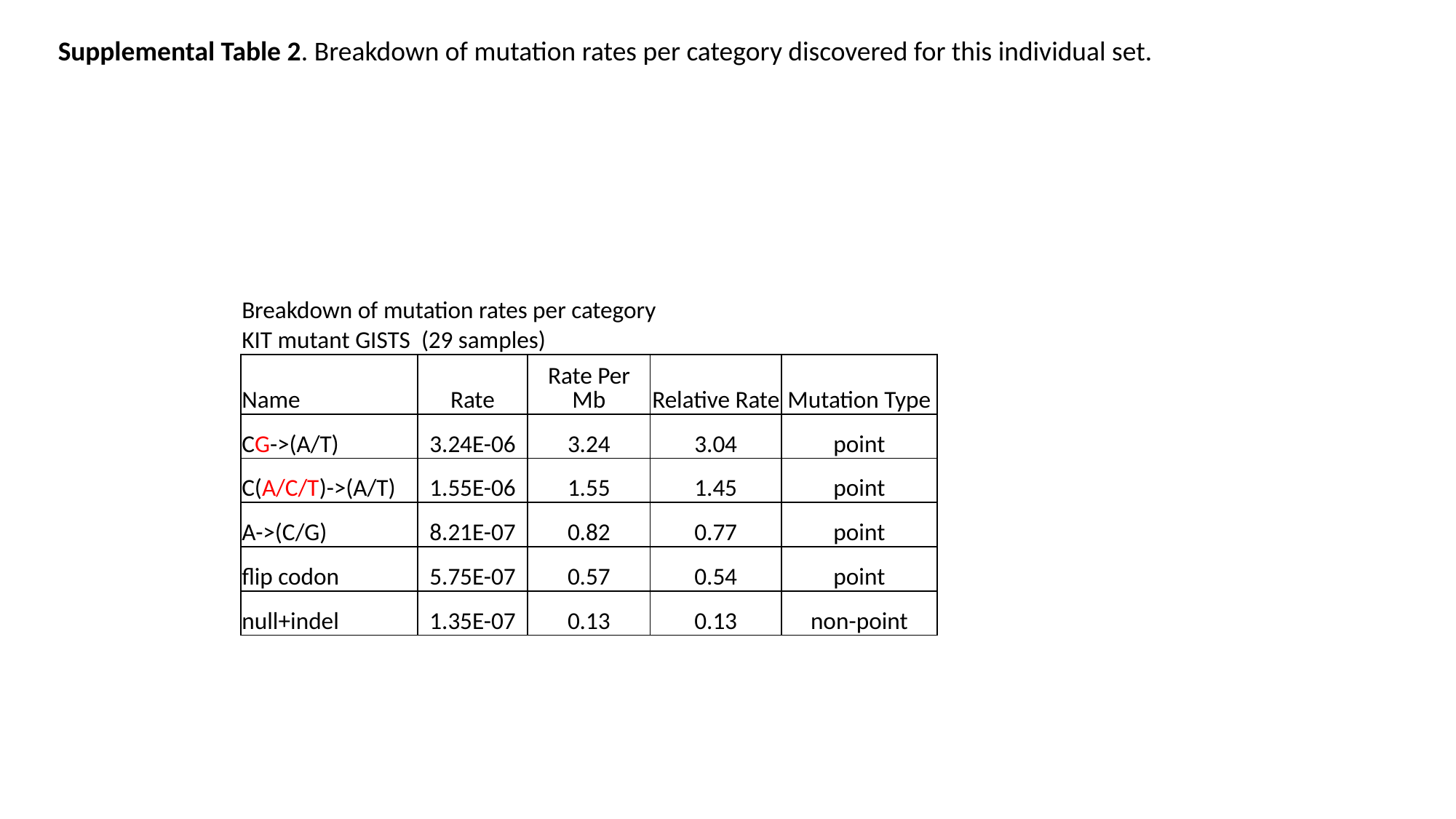

Supplemental Table 2. Breakdown of mutation rates per category discovered for this individual set.
| Breakdown of mutation rates per category | | | | |
| --- | --- | --- | --- | --- |
| KIT mutant GISTS (29 samples) | | | | |
| Name | Rate | Rate Per Mb | Relative Rate | Mutation Type |
| CG->(A/T) | 3.24E-06 | 3.24 | 3.04 | point |
| C(A/C/T)->(A/T) | 1.55E-06 | 1.55 | 1.45 | point |
| A->(C/G) | 8.21E-07 | 0.82 | 0.77 | point |
| flip codon | 5.75E-07 | 0.57 | 0.54 | point |
| null+indel | 1.35E-07 | 0.13 | 0.13 | non-point |

## Slide 5
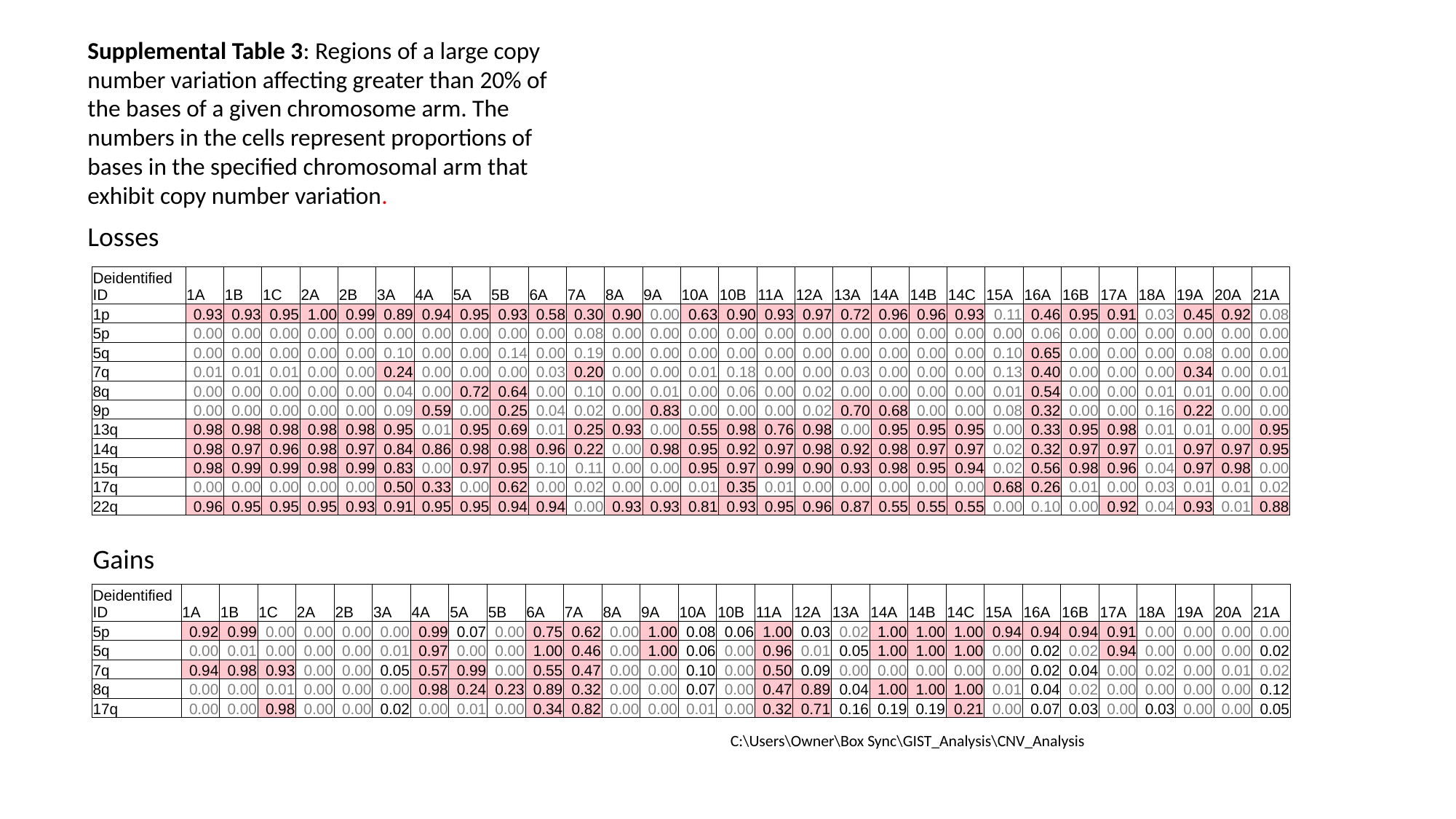

Supplemental Table 3: Regions of a large copy number variation affecting greater than 20% of the bases of a given chromosome arm. The numbers in the cells represent proportions of bases in the specified chromosomal arm that exhibit copy number variation.
Losses
| Deidentified ID | 1A | 1B | 1C | 2A | 2B | 3A | 4A | 5A | 5B | 6A | 7A | 8A | 9A | 10A | 10B | 11A | 12A | 13A | 14A | 14B | 14C | 15A | 16A | 16B | 17A | 18A | 19A | 20A | 21A |
| --- | --- | --- | --- | --- | --- | --- | --- | --- | --- | --- | --- | --- | --- | --- | --- | --- | --- | --- | --- | --- | --- | --- | --- | --- | --- | --- | --- | --- | --- |
| 1p | 0.93 | 0.93 | 0.95 | 1.00 | 0.99 | 0.89 | 0.94 | 0.95 | 0.93 | 0.58 | 0.30 | 0.90 | 0.00 | 0.63 | 0.90 | 0.93 | 0.97 | 0.72 | 0.96 | 0.96 | 0.93 | 0.11 | 0.46 | 0.95 | 0.91 | 0.03 | 0.45 | 0.92 | 0.08 |
| 5p | 0.00 | 0.00 | 0.00 | 0.00 | 0.00 | 0.00 | 0.00 | 0.00 | 0.00 | 0.00 | 0.08 | 0.00 | 0.00 | 0.00 | 0.00 | 0.00 | 0.00 | 0.00 | 0.00 | 0.00 | 0.00 | 0.00 | 0.06 | 0.00 | 0.00 | 0.00 | 0.00 | 0.00 | 0.00 |
| 5q | 0.00 | 0.00 | 0.00 | 0.00 | 0.00 | 0.10 | 0.00 | 0.00 | 0.14 | 0.00 | 0.19 | 0.00 | 0.00 | 0.00 | 0.00 | 0.00 | 0.00 | 0.00 | 0.00 | 0.00 | 0.00 | 0.10 | 0.65 | 0.00 | 0.00 | 0.00 | 0.08 | 0.00 | 0.00 |
| 7q | 0.01 | 0.01 | 0.01 | 0.00 | 0.00 | 0.24 | 0.00 | 0.00 | 0.00 | 0.03 | 0.20 | 0.00 | 0.00 | 0.01 | 0.18 | 0.00 | 0.00 | 0.03 | 0.00 | 0.00 | 0.00 | 0.13 | 0.40 | 0.00 | 0.00 | 0.00 | 0.34 | 0.00 | 0.01 |
| 8q | 0.00 | 0.00 | 0.00 | 0.00 | 0.00 | 0.04 | 0.00 | 0.72 | 0.64 | 0.00 | 0.10 | 0.00 | 0.01 | 0.00 | 0.06 | 0.00 | 0.02 | 0.00 | 0.00 | 0.00 | 0.00 | 0.01 | 0.54 | 0.00 | 0.00 | 0.01 | 0.01 | 0.00 | 0.00 |
| 9p | 0.00 | 0.00 | 0.00 | 0.00 | 0.00 | 0.09 | 0.59 | 0.00 | 0.25 | 0.04 | 0.02 | 0.00 | 0.83 | 0.00 | 0.00 | 0.00 | 0.02 | 0.70 | 0.68 | 0.00 | 0.00 | 0.08 | 0.32 | 0.00 | 0.00 | 0.16 | 0.22 | 0.00 | 0.00 |
| 13q | 0.98 | 0.98 | 0.98 | 0.98 | 0.98 | 0.95 | 0.01 | 0.95 | 0.69 | 0.01 | 0.25 | 0.93 | 0.00 | 0.55 | 0.98 | 0.76 | 0.98 | 0.00 | 0.95 | 0.95 | 0.95 | 0.00 | 0.33 | 0.95 | 0.98 | 0.01 | 0.01 | 0.00 | 0.95 |
| 14q | 0.98 | 0.97 | 0.96 | 0.98 | 0.97 | 0.84 | 0.86 | 0.98 | 0.98 | 0.96 | 0.22 | 0.00 | 0.98 | 0.95 | 0.92 | 0.97 | 0.98 | 0.92 | 0.98 | 0.97 | 0.97 | 0.02 | 0.32 | 0.97 | 0.97 | 0.01 | 0.97 | 0.97 | 0.95 |
| 15q | 0.98 | 0.99 | 0.99 | 0.98 | 0.99 | 0.83 | 0.00 | 0.97 | 0.95 | 0.10 | 0.11 | 0.00 | 0.00 | 0.95 | 0.97 | 0.99 | 0.90 | 0.93 | 0.98 | 0.95 | 0.94 | 0.02 | 0.56 | 0.98 | 0.96 | 0.04 | 0.97 | 0.98 | 0.00 |
| 17q | 0.00 | 0.00 | 0.00 | 0.00 | 0.00 | 0.50 | 0.33 | 0.00 | 0.62 | 0.00 | 0.02 | 0.00 | 0.00 | 0.01 | 0.35 | 0.01 | 0.00 | 0.00 | 0.00 | 0.00 | 0.00 | 0.68 | 0.26 | 0.01 | 0.00 | 0.03 | 0.01 | 0.01 | 0.02 |
| 22q | 0.96 | 0.95 | 0.95 | 0.95 | 0.93 | 0.91 | 0.95 | 0.95 | 0.94 | 0.94 | 0.00 | 0.93 | 0.93 | 0.81 | 0.93 | 0.95 | 0.96 | 0.87 | 0.55 | 0.55 | 0.55 | 0.00 | 0.10 | 0.00 | 0.92 | 0.04 | 0.93 | 0.01 | 0.88 |
Gains
| Deidentified ID | 1A | 1B | 1C | 2A | 2B | 3A | 4A | 5A | 5B | 6A | 7A | 8A | 9A | 10A | 10B | 11A | 12A | 13A | 14A | 14B | 14C | 15A | 16A | 16B | 17A | 18A | 19A | 20A | 21A |
| --- | --- | --- | --- | --- | --- | --- | --- | --- | --- | --- | --- | --- | --- | --- | --- | --- | --- | --- | --- | --- | --- | --- | --- | --- | --- | --- | --- | --- | --- |
| 5p | 0.92 | 0.99 | 0.00 | 0.00 | 0.00 | 0.00 | 0.99 | 0.07 | 0.00 | 0.75 | 0.62 | 0.00 | 1.00 | 0.08 | 0.06 | 1.00 | 0.03 | 0.02 | 1.00 | 1.00 | 1.00 | 0.94 | 0.94 | 0.94 | 0.91 | 0.00 | 0.00 | 0.00 | 0.00 |
| 5q | 0.00 | 0.01 | 0.00 | 0.00 | 0.00 | 0.01 | 0.97 | 0.00 | 0.00 | 1.00 | 0.46 | 0.00 | 1.00 | 0.06 | 0.00 | 0.96 | 0.01 | 0.05 | 1.00 | 1.00 | 1.00 | 0.00 | 0.02 | 0.02 | 0.94 | 0.00 | 0.00 | 0.00 | 0.02 |
| 7q | 0.94 | 0.98 | 0.93 | 0.00 | 0.00 | 0.05 | 0.57 | 0.99 | 0.00 | 0.55 | 0.47 | 0.00 | 0.00 | 0.10 | 0.00 | 0.50 | 0.09 | 0.00 | 0.00 | 0.00 | 0.00 | 0.00 | 0.02 | 0.04 | 0.00 | 0.02 | 0.00 | 0.01 | 0.02 |
| 8q | 0.00 | 0.00 | 0.01 | 0.00 | 0.00 | 0.00 | 0.98 | 0.24 | 0.23 | 0.89 | 0.32 | 0.00 | 0.00 | 0.07 | 0.00 | 0.47 | 0.89 | 0.04 | 1.00 | 1.00 | 1.00 | 0.01 | 0.04 | 0.02 | 0.00 | 0.00 | 0.00 | 0.00 | 0.12 |
| 17q | 0.00 | 0.00 | 0.98 | 0.00 | 0.00 | 0.02 | 0.00 | 0.01 | 0.00 | 0.34 | 0.82 | 0.00 | 0.00 | 0.01 | 0.00 | 0.32 | 0.71 | 0.16 | 0.19 | 0.19 | 0.21 | 0.00 | 0.07 | 0.03 | 0.00 | 0.03 | 0.00 | 0.00 | 0.05 |
C:\Users\Owner\Box Sync\GIST_Analysis\CNV_Analysis
