# Supplementary material for: Genomic aberrations in cell cycle genes predict progression of KIT-mutant gastrointestinal stromal tumors (GISTs)
Source: Clin Sarcoma Res. 2019 Mar 5;9:3. doi: 10.1186/s13569-019-0112-7 (PMC6399846; doi:10.1186/s13569-019-0112-7)
Supplement: Supplementary file 6 — Additional file 6: Table S8. Multi-variate analysis of clinically characterized GIST. [file 13569_2019_112_MOESM6_ESM.pptx]

## Slide 1
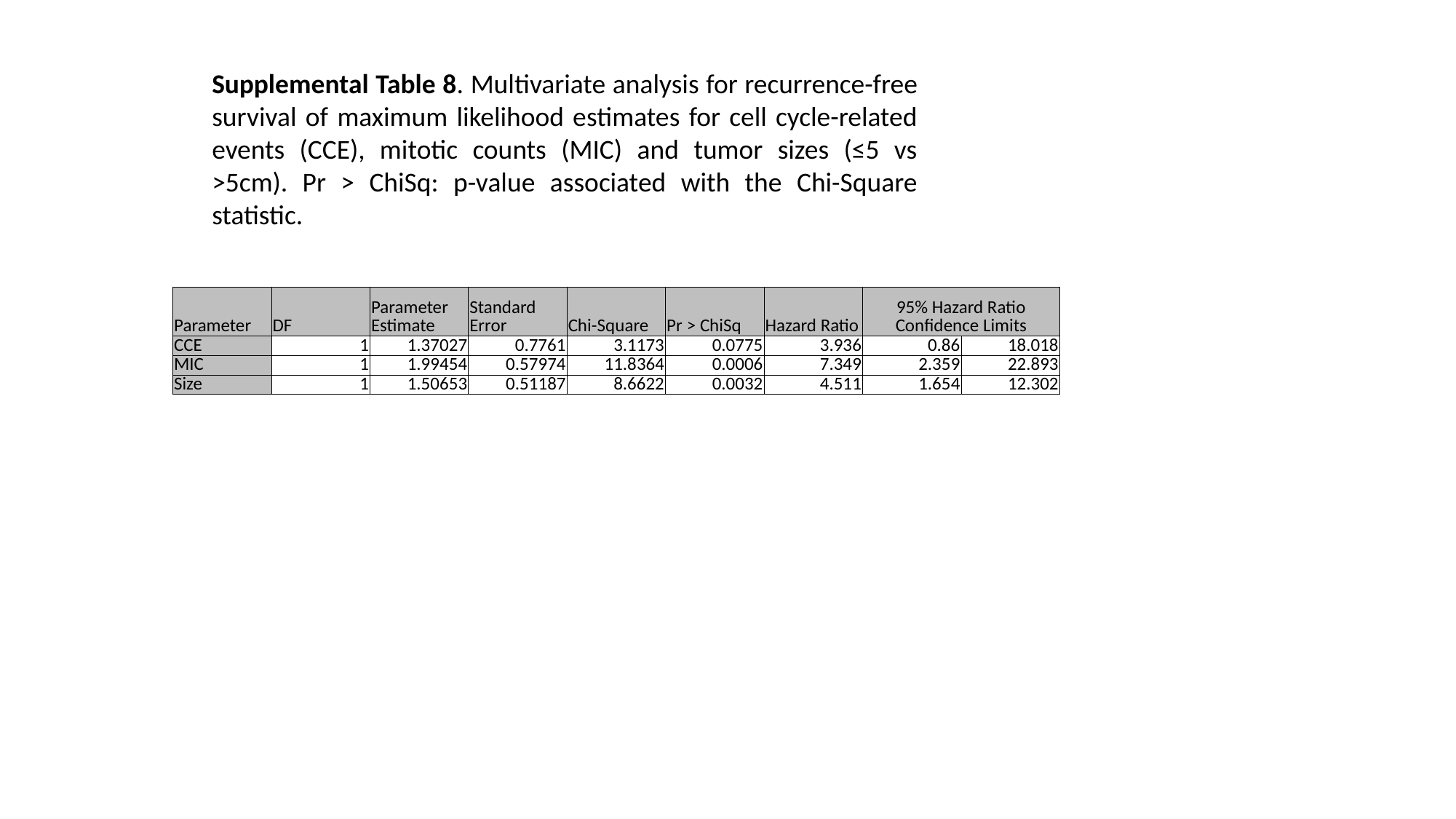

Supplemental Table 8. Multivariate analysis for recurrence-free survival of maximum likelihood estimates for cell cycle-related events (CCE), mitotic counts (MIC) and tumor sizes (≤5 vs >5cm). Pr > ChiSq: p-value associated with the Chi-Square statistic.
| Parameter | DF | Parameter Estimate | Standard Error | Chi-Square | Pr > ChiSq | Hazard Ratio | 95% Hazard Ratio Confidence Limits | |
| --- | --- | --- | --- | --- | --- | --- | --- | --- |
| CCE | 1 | 1.37027 | 0.7761 | 3.1173 | 0.0775 | 3.936 | 0.86 | 18.018 |
| MIC | 1 | 1.99454 | 0.57974 | 11.8364 | 0.0006 | 7.349 | 2.359 | 22.893 |
| Size | 1 | 1.50653 | 0.51187 | 8.6622 | 0.0032 | 4.511 | 1.654 | 12.302 |
